# The Skp1 Homologs SKR-1/2 Are Required for the *Caenorhabditis elegans* SKN-1 Antioxidant/Detoxification Response Independently of p38 MAPK

**DOI:** 10.1371/journal.pgen.1006361

**Published:** 2016-10-24

**Authors:** Cheng-Wei Wu, Andrew Deonarine, Aaron Przybysz, Kevin Strange, Keith P. Choe

**Affiliations:** 1 Department of Biology and Genetics Institute, University of Florida, Gainesville, FL 32611, USA; 2 Department of Cell Biology, Microbiology, and Molecular Biology, University of South Florida, Tampa, FL, 33620; 3 Department of Anesthesiology, University of Michigan, Ann Arbor, MI 48109; 4 The MDI Biological Laboratory, Salisbury Cove, ME 04672; The University of Texas Health Science Center at Houston, UNITED STATES

## Abstract

SKN-1/Nrf are the primary antioxidant/detoxification response transcription factors in animals and they promote health and longevity in many contexts. SKN-1/Nrf are activated by a remarkably broad-range of natural and synthetic compounds and physiological conditions. Defining the signaling mechanisms that regulate SKN-1/Nrf activation provides insights into how cells coordinate responses to stress. Nrf2 in mammals is regulated in part by the redox sensor repressor protein named Keap1. In *C*. *elegans*, the p38 MAPK cascade in the intestine activates SKN-1 during oxidative stress by promoting its nuclear accumulation. Interestingly, we find variation in the kinetics of p38 MAPK activation and tissues with SKN-1 nuclear accumulation among different pro-oxidants that all trigger strong induction of SKN-1 target genes. Using genome-wide RNAi screening, we identify new genes that are required for activation of the core SKN-1 target gene *gst-4* during exposure to the natural pro-oxidant juglone. Among 10 putative activators identified in this screen was *skr-1/2*, highly conserved homologs of yeast and mammalian Skp1, which function to assemble protein complexes. Silencing of *skr-1/2* inhibits induction of SKN-1 dependent detoxification genes and reduces resistance to pro-oxidants without decreasing p38 MAPK activation. Global transcriptomics revealed strong correlation between genes that are regulated by SKR-1/2 and SKN-1 indicating a high degree of specificity. We also show that SKR-1/2 functions upstream of the WD40 repeat protein WDR-23, which binds to and inhibits SKN-1. Together, these results identify a novel p38 MAPK independent signaling mechanism that activates SKN-1 *via* SKR-1/2 and involves WDR-23.

## Author Summary

Oxidative stress is the result of imbalanced control of reactive oxidative species in cells, is a common occurrence during aerobic metabolism, and must be managed to limit cellular damage and disease. Many details about the signaling mechanisms utilized by animal cells in response to pro-oxidants remain to be discovered. We provide evidence that the signaling mechanisms that activate SKN-1, a master regulator of detoxification genes and aging in the model organism *C*. *elegans*, may vary in response to different oxidative stressors. From a genome-wide genetic screen, we identify highly conserved genes *skr-1/2* as central regulators of the gene response to pro-oxidants. During exposure to oxidants, SKR-1/2 function upstream from SKN-1 and a direct SKN-1 repressor named WDR-23. These results provide new insights into our understanding of SKN-1 regulation and lay the foundation for future studies to define in detail novel signaling pathways that respond to pro-oxidants.

## Introduction

Reactive small molecules are common in natural environments and are produced as byproducts of oxygen metabolism. Reactive small molecules in excess can cause oxidative damage with widespread detrimental effects, but also function as signaling molecules for normal physiological processes [1]. Appropriate response to and regulation of these compounds is crucial as aberrant accumulation has been implicated in early onset of aging along with many pathological states that include metabolic syndromes, neurological disorders, and cancer [2,3,4]. In *C*. *elegans*, the cap ‘n’ collar transcription factor family member SKN-1 is homologous to mammalian Nrf2 and functions to promote longevity and resistance to a wide range of environmental stressors [5].

In response to a wide-range of reactive small molecules, SKN-1/Nrf transcription factors translocate into the nucleus and bind to response elements in target genes to activate a conserved detoxification response [6,7,8]. In mammals, Keap1 represses basal Nrf2 activity through a direct interaction that promotes ubiquitylation and degradation, and there is strong support for a model in which small molecules directly react with Keap1 releasing Nrf2 from repression [6,7,8]. Additional Keap1-independent signaling mechanisms exist that are less-defined [9].

Genetic tractability and the conserved nature of the SKN-1/Nrf response have made *C*. *elegans* an important model for regulation of this pathway [5]. *C*. *elegans* has also been instrumental for defining SKN-1 as a central determinant of aging and longevity [10,11,12] and is being used to understand the role of SKN-1 in antiparasitic drug resistance [13,14,15]. Although *C*. *elegans* lacks a close Keap1 homolog, it is repressed under basal conditions by an analogous mechanism *via* the WD40 repeat protein WDR-23, which binds to and inhibits SKN-1 [15,16]. The protein kinases AKT-1/2, SGK-1, and GSK-3 also function to inhibit SKN-1 under basal conditions [11,17]. A number of protein kinases have been implicated in activation of SKN-1 (MKK-4, IKKɛ-1, NEKL-2, and PHDK-2), although it is not known if any of these regulate SKN-1 directly [18]. During oxidative stress, the p38 MAPK signaling cascade directly phosphorylates and promotes nuclear accumulation of SKN-1 in cells of the intestine [19], a tissue thought to be a primary site for detoxification; p38 MAPK is also required for activation of SKN-1 in the intestine during infection [20,21]. A recent study demonstrated that TIR-1, Toll/interleukin-1 receptor domain protein, functions upstream from p38 MAPK during exposure to an oxidant [22]. Although protein kinases, particularly p38 MAPK, are clearly important, it is not known if this one mechanism is responsible for activation of SKN-1 by all the diverse reactive small molecules known to strongly activate the pathway.

We demonstrate here that the kinetics of p38 MAPK activation and tissues with SKN-1::GFP accumulation vary with different pro-oxidants that all elicit a strong SKN-1 dependent detoxification response. Using genome-wide RNAi screening, we identified SKR-1/2 as required for the core SKN-1 transcriptional response to diverse pro-oxidant compounds. SKR-1/2 are highly conserved orthologues of Skp1, a component of many protein complexes including the Skp-Cullin-F box ubiquitin ligase (SCF) that regulates cell cycle progression and differentiation [23,24]. Loss of *skr-1/2* strongly and specifically attenuates induction of SKN-1 dependent genes independent of p38 MAPK signaling and reduces survival of pro-oxidants. SKR-1/2 functions upstream of WDR-23 and influences the accumulation of a WDR-23::GFP fusion protein in nuclei. We hypothesize that this newly identified pathway regulates SKN-1 activity by modulating WDR-23 function.

## Results

### Kinetics of p38 MAPK activation varies among pro-oxidants that induce a SKN-1 dependent detoxification response

**Fig 1:**
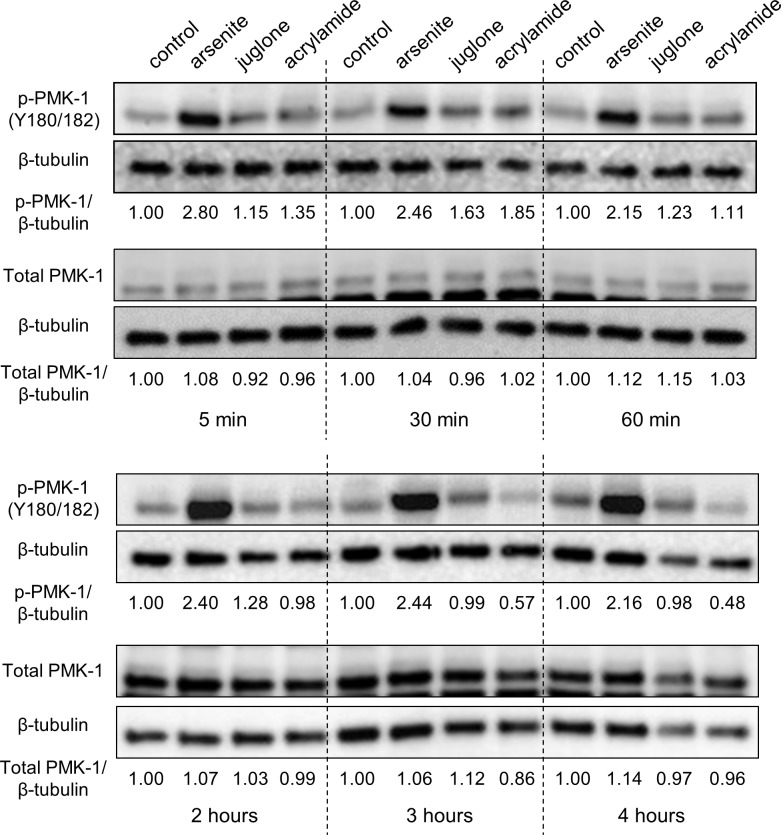
Kinetics of PMK-1 activation. Relative level of PMK-1 phosphorylation at the kinase activating residues (Y180/182) and total PMK-1 protein levels in L4/YA worms treated with 5 mM sodium arsenite, 38 μM juglone, or 7 mM acrylamide for up to four hours. Band intensities relative to β-tubulin are given below each pair of blots with control arbitrarily set to 1.

SKN-1 activation has been shown to be mediated through direct phosphorylation by PMK-1 (p38 MAPK) in response to oxidative stress induced by arsenite and during pathogen infection (*Pseudomonas aeruginosa*) [19,20,21]. SKN-1 residues phosphorylated by PMK-1 are required for nuclear accumulation, a step sometimes correlated with induction of SKN-1 dependent detoxification genes. Arsenite, paraquat, juglone, and acrylamide are a diverse set of small molecules that all strongly activate SKN-1 dependent detoxification genes [16,19,25]. To test if PMK-1 responded similarly to these different compounds, we measured phosphorylation of PMK-1 at its kinase activation residue (Y180/182) over a four hour time course following exposure to arsenite, juglone, and acrylamide (Fig 1). Arsenite increased PMK-1 phosphorylation levels strongly at all time points, and juglone and acrylamide caused smaller transient increases during short term exposure (5–60 min). Interestingly, PMK-1 phosphorylation levels decreased with acrylamide after 3 and 4 h (Fig 1).

**Fig 2:**
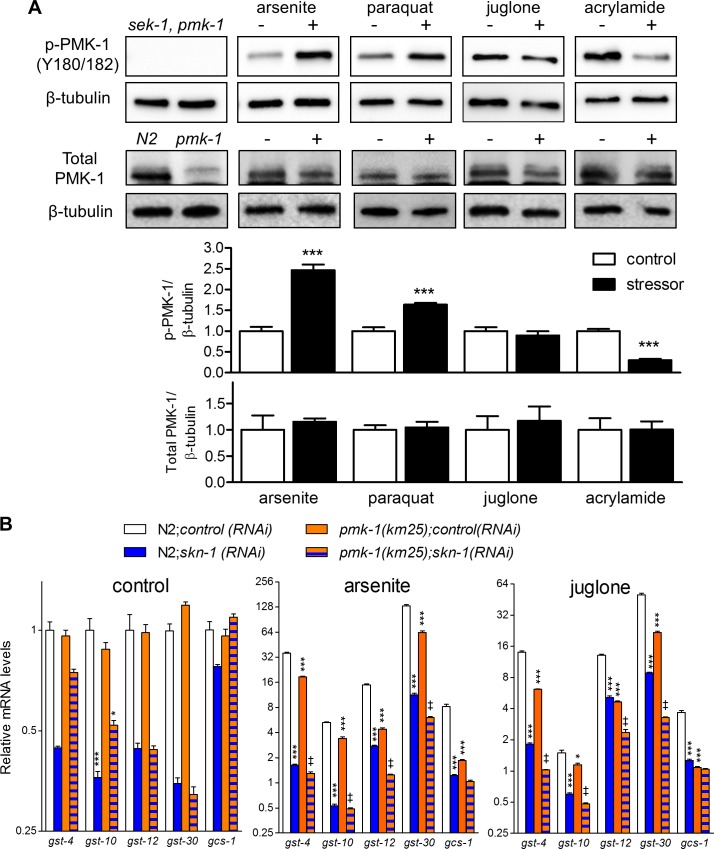
PMK-1 activation and its requirement for SKN-1 transcriptional responses. (A) Relative level of PMK-1 phosphorylation at the kinase activating residues (Y180/182) and total PMK-1 protein levels in L4/YA worms treated with 5 mM sodium arsenite for 1 h, 35 mM paraquat for 2 h, 38 μM juglone for 3 h, or 7 mM acrylamide for 4 h. PMK-1 phosphorylation and protein levels were normalized to β-tubulin, which was detected on the same blot after stripping; histograms represent mean plus standard error of densitometry from *n* = 4 replicates of ~1,000 worms. ***P<0.001 compared to corresponding control as determined by Student’s T-test. Representative Western blot images are shown. (B) Fold changes in *gst-4*, *gst-10*, *gst-12*, *gst-30*, and *gcs-1* mRNA levels relative to N2 control in L4/YA N2 wildtype and *pmk-1*(*km25*) mutant worms treated with 5 mM sodium arsenite or 38 μM juglone for 1 h, worms were fed either control RNAi or *skn-1* RNAi from L1. Histograms represent mean plus standard error of *n* = 4 replicates of 200–300 worms. All genes were induced significantly by arsenite or juglone (P<0.001); *P<0.05, *** P<0.001 compared to N2;*control(RNAi)*, ‡ P<0.001 compared to *pmk-1(km25);control(RNAi)*.

We also measured phosphorylation of PMK-1 with replicates to confirm effects at specific time points and to test paraquat; doses and durations used were all sub-lethal (S1 Fig) yet strongly activate SKN-1 dependent genes (5 mM arsenite for 1 h, 35 mM paraquat for 2 h, 38 μM juglone for 3 h, and 7 mM acrylamide for 4 h). An increase in phosphorylation of PMK-1 was observed with arsenite and paraquat and a decrease was confirmed for 4 h of acrylamide exposure (Fig 2A). Total levels of PMK-1 were not altered with any treatment or duration (Figs 1 and 2A), indicating that changes to p-PMK-1 were at the posttranslational level. Given that we used whole animal lysates, these results may not reflect PMK-1 phosphorylation kinetics in all tissues. However, our results do demonstrate that the overall patterns of PMK-1 phosphorylation can vary between conditions that all strongly activate SKN-1.

We next used real-time RT-PCR (qPCR) in deletion mutants of *pmk-1* with and without *skn-1(RNAi)* to assess the requirement of the p38 MAPK pathway after exposure to 5 mM arsenite or 38 μM juglone for 1 h. As expected, five detoxification genes directly regulated by SKN-1 were strongly activated by both compounds in N2 wild-type worms and this was partially dependent on *pmk-1* and largely dependent on *skn-1* (Fig 2B). Although all four *gst* detoxification genes were partially dependent on *pmk-1*, they were still induced. Given that the *pmk-1*(*km25*) allele we used is a deletion considered to be null, these results suggest that there are mechanisms that can compensate for loss of PMK-1 in these contexts (e.g., PMK-1 paralogs or other pathways).

### SKN-1 accumulation in nuclei can be decoupled from induction of SKN-1 dependent detoxification genes

**Fig 3:**
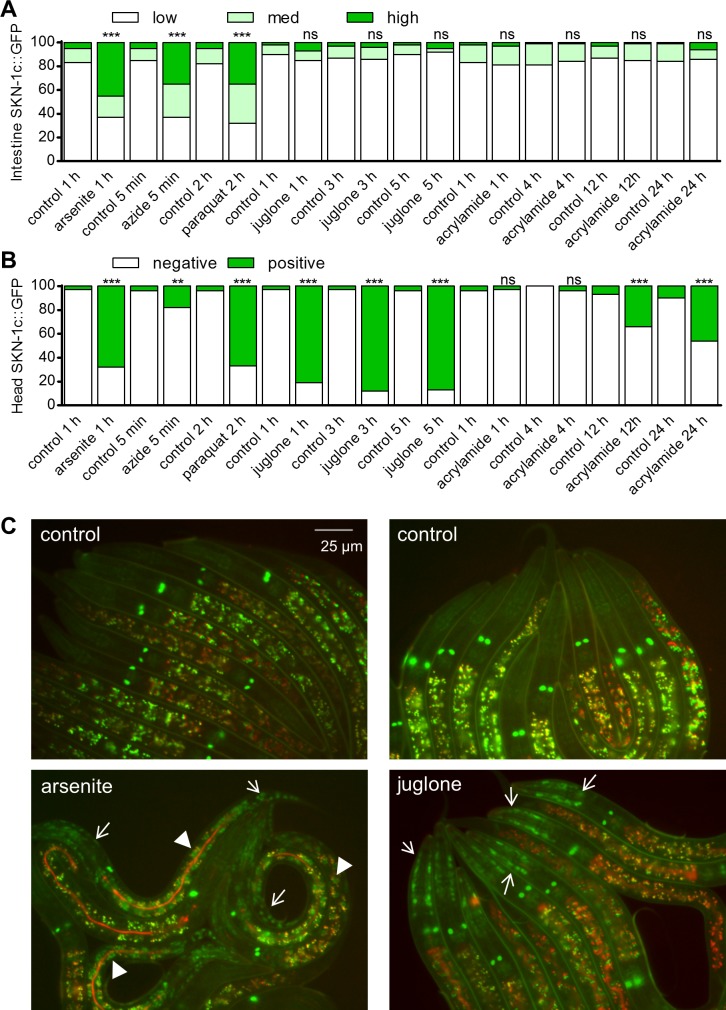
SKN-1 is localized to multiple tissues in a stress dependent manner. Animals integrated with a SKN-1b/c::GFP transgene were treated with 5 mM sodium arsenite, 5 mM sodium azide, 35 mM paraquat, 38 μM juglone, or 7 mM acrylamide. Accumulation of intestinal (A) and head (B) SKN-1b/c::GFP were scored separately. *n* = 60–100 worms from 3 independent trials. ***P<0.001 from corresponding controls as determined by Chi-Square tests. For intestinal SKN-1b/c::GFP, low refers to little to no SKN-1b/c::GFP, medium refers to SKN-1b/c::GFP observed only at the anterior or posterior of the intestine, and high refers to SKN-1b/c::GFP observed throughout the intestine. For head SKN-1b/c::GFP, negative refers to no observation of GFP signals in the head region, SKN-1b/c::GFP positive refers to GFP signals observed throughout the head region. (C) Representative fluorescence micrographs are shown for arsenite and juglone. Arrows mark head GFP and arrowheads mark intestinal nuclei.

Arsenite induces strong nuclear accumulation of SKN-1b/c::GFP fusion proteins in the intestine, which is dependent on the p38 MAPK pathway [19]. Although SKN-1 protein accumulation in nuclei is thought to be one mechanism of pathway activation [19], genetic and environmental conditions have been identified that activate SKN-1 target genes without causing detectable SKN-1b/c::GFP accumulation implicating other yet-to-be defined regulatory mechanisms [26,27,28]. We scored the percentage of worms with three levels of intestinal SKN-1b/c::GFP nuclear accumulation with arsenite, azide, paraquat, juglone, and acrylamide exposure to determine if SKN-1 accumulation varies with SKN-1 inducer. The LD001 strain we used expresses fusion proteins of SKN-1c and b variants, but not the longer SKN-1a variant [6]. Consistent with previous reports, we found that 5 mM arsenite, 5 mM azide, and 35 mM paraquat induced high levels of nuclear SKN-1b/c::GFP localization in the intestine (Fig 3A). Conversely, nuclear SKN-1b/c::GFP was not detected in the intestine with short (5–15 min, S2 Fig) or long-term (up to 5 h of 38 μM juglone or 24 h of 7 mM acrylamide, Fig 3A) exposure to juglone or acrylamide even though these treatments strongly activate SKN-1 dependent detoxification genes in the same tissue [16,25] (S3 Fig).

Scoring of stress-inducible SKN-1b/c::GFP localization is typically limited to the intestine, which is credited with being the primary site of detoxification and has the largest nuclei that are readily visible. However, we and others [25] observe strong induction of SKN-1 dependent detoxification gene reporters in other tissues, particularly the hypodermis (S3 Fig). Careful observation revealed SKN-1b/c::GFP accumulation in nuclei throughout worms exposed to arsenite (Fig 3B and 3C and S4A Fig); these other nuclei are most obvious in the head and tail regions, which have less autofluorescence than areas around the intestine. No accumulation was observed in intestine, head, or tail regions of worms fed *skn-1* dsRNA (S4A Fig) verifying specificity of the signal. We also scored nuclear accumulation with a transgene that covers all forms of SKN-1 including the long SKN-1a variant, *skn-1op*::*GFP* [11]. As shown in S4B Fig, SKN-1op::GFP generally responded similar to SKN-1b/c::GFP with no intestinal accumulation with juglone.

**Fig 4:**
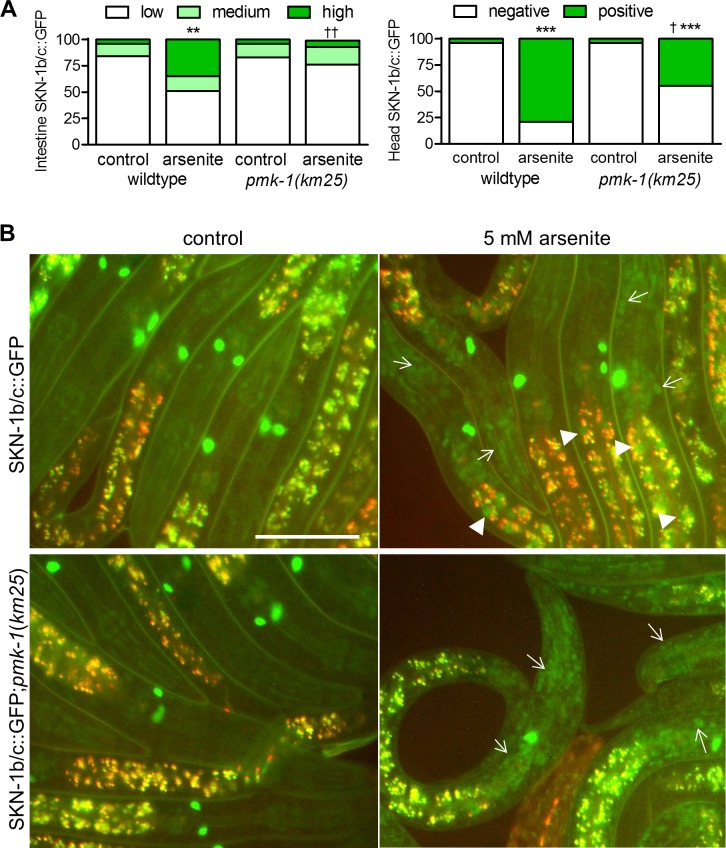
SKN-1b/c::GFP can accumulate in the head without *pmk-1*. (A) Scoring of SKN-1b/c::GFP was conducted as in Fig 2. **P<0.01 and ***P<0.001 relative to the corresponding controls without arsenite as determined by Chi-Square tests, †P<0.05 and ††P<0.01 relative to the arsenite exposed wildtype worms as determined by Chi-Square tests, *n =* 51–92. (B) Representative images of SKN-1b/c::GFP. Arrows mark head GFP and arrowheads mark intestinal nuclei.

The head nuclei SKN-1b/c::GFP signals were also consistently induced by azide, paraquat, and juglone (Fig 3B and 3C); head nuclei SKN-1b/c::GFP signals were also induced by acrylamide, but this took several hours. Although identification of all tissues with SKN-1b/c::GFP is difficult, at least some of the signal appears to be in hypodermal cell nuclei based on location and morphology (S4C Fig) when compared to images of hypodermal specific markers [29]. The *pmk-1(km25)* allele was crossed into the SKN-1b/c::GFP strain to test the requirement of p38 MAPK for the accumulation of hypodermal SKN-1. As expected, SKN-1b/c::GFP failed to accumulate in the intestine of *pmk-1* worms under arsenite exposure (Fig 4). Alternatively, loss of *pmk-1* only slightly reduced the number of worms with SKN-1b/c::GFP accumulation in the head region (Fig 4).

To determine the physiological role of SKN-1 in the hypodermis and intestine, we next measured the effects of hypodermis and intestine-specific *skn-1(RNAi)* on juglone survival. As shown in S5 Fig, loss of *skn-1* from either tissue reduced survival consistent with *skn-1* mediating resistance in both tissues even though SKN-1::GFP accumulation is not obvious in the intestine during exposure to juglone. Therefore, juglone and acrylamide are able to activate a robust SKN-1 dependent detoxification gene response without detectable SKN-1::GFP nuclear accumulation in the intestine, and SKN-1b/c::GFP accumulation during stress can be partially decoupled from *pmk-1* in tissues other than the intestine.

### Genome-wide screen for new regulators of SKN-1 target gene activation

**Fig 5:**
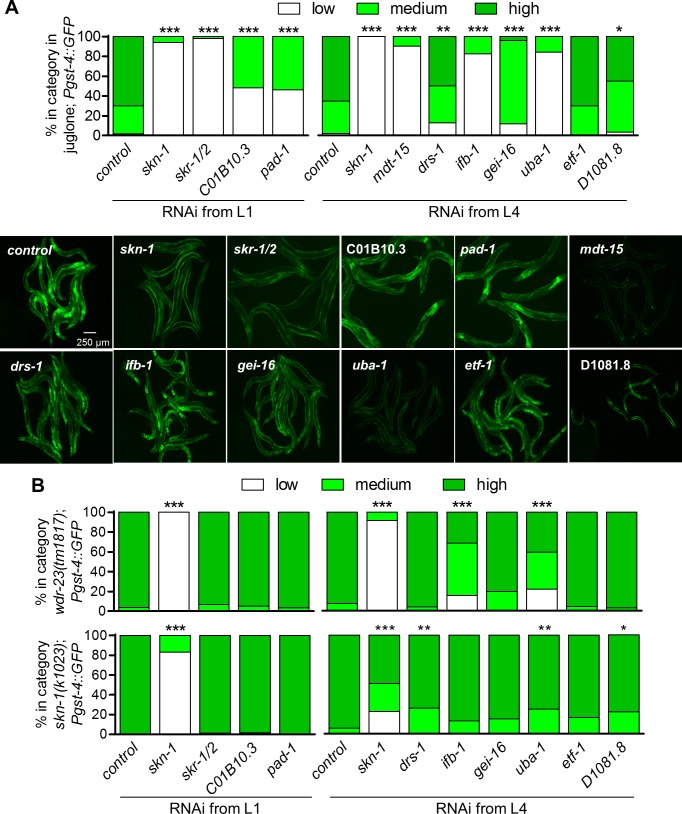
New *gst-4* regulators identified through genome wide RNAi screening function upstream of *wdr-23* and *skn-1*. Animals integrated with *Pgst-4*::*GFP* were fed bacteria producing dsRNA to positive hits obtained from the RNAi screen and exposed to juglone. (A) *Pgst-4*::*GFP* fluorescence was scored and representative images are shown. For *Pgst-4*::*GFP* scoring, low refers to little to no GFP signals observed throughout the worm, medium refers to GFP signals observed at the anterior and posterior ends of the worm, and high refers to GFP signals observed throughout the body. *n* = 50–118 worms. (B) *Pgst-4*::*GFP* reporter scoring in either a *wdr-23* loss of function (*tm1817*) or a *skn-1* gain of function allele (*k1023*) mutant background. *n* = 54–75 worms. *P<0.05, **P<0.01 and ***P<0.001 relative to corresponding control as determined by Chi-Square tests; note that *pad-1(RNAi)* in *skn-1(k1023)* could not be tested for significance because it had zero worms with low or medium fluorescence.

To identify new regulators of the SKN-1 detoxification response, we utilized a *C*. *elegans* strain harboring an integrated *Pgst-4*::*GFP* transcriptional reporter in a genome-wide RNAi screen for genes that regulate *gst-4* expression during exposure to juglone. *gst-4* is a phase 2 detoxification gene regulated directly by SKN-1 under numerous conditions, and it has been shown to be a reliable reporter for SKN-1 transcriptional activity [25,27,30,31]. We screened approximately 19,000 dsRNA clones and identified 10 genes that when silenced consistently reduced juglone-induced *Pgst-4*::*GFP* fluorescence. RNAi of seven of these genes caused developmental defects or general sickness. After retesting these by initiating silencing at the L4 larval stage rather than L1 to bypass developmental requirements, there were a total of six RNAi clones that strongly reduced *Pgst-4*::*GFP* fluorescence. These were *skr-1/2*, C01B10.3, *pad-1*, *mdt-15*, *ifb-1*, and *uba-1* (Fig 5A); *mdt-15* has previously been reported to function with SKN-1 [32]. We tested the candidate novel regulators with two other stressors to assess specificity. None of the novel *gst-4* regulators had a significant effect on induction of a heat shock reporter, *Phsp-16*.*2*::*GFP* (S6A Fig), but *ifb-1*, *uba-1*, *etf-1*, and D1081.8 were required for induction of an osmotic stress reporter, *Pgpdh-1*::*RFP* (S6B Fig).

We next tested the candidate genes for genetic interactions with *wdr-23* and *skn-1* using strains carrying the *Pgst-4*::*GFP* reporter and a *wdr-23*(*tm1817)* loss of function allele or a *skn-1*(*k1023*) gain of function allele [10]. Both strains have constitutive activation of *Pgst-4*::*GFP* that is suppressed by *skn-1(RNAi)*. dsRNA targeting the candidates had little or no effect on *Pgst-4*::*GFP* with the exception of *ifb-1* and *uba-1* in *wdr-23*(*tm1817*) (Fig 5B); although not confirmed in these experiments, *skr-1/2(RNAi)* consistently causes a partial embryonic lethality phenotype. These findings suggest that *skr-1/2*, C01B10.3, and *pad-1* likely function upstream of *skn-1* and *wdr-23*.

### *skr-1/2* is required for induction of *skn-1* dependent detoxification genes by diverse compounds

*skr-1/2(RNAi)* (Skp-related) caused the strongest inhibition of *Pgst-4*::*GFP* with juglone, and most closely resembled the pattern observed for *skn-1(RNAi)* (Fig 5A). SKR-1/2 was previously shown to be required for longevity extension in *daf-2* insulin and IGF-1-like receptor mutants [33], but has not previously been reported to function as a regulator of stress responses. Because the neighboring *skr-1* and *skr-2* genes are recent duplications that are 83% identical at the nucleotide level, *skr-1* dsRNA likely silences both *skr-1* and *skr-2* [24]. Consistent with this, *skr-1* dsRNA reduces both *skr-1* and *skr-2* mRNA (S1 Table) and *skr-2* dsRNA has the same effect on *Pgst-4*::*GFP* as *skr-1* dsRNA (S7A Fig). We therefore refer to ‘*skr-1/2(RNAi)’* when using *skr-1* dsRNA.

*C*. *elegans* has a total of 21 SKR proteins that are orthologous to the single yeast and mammalian Skp1 proteins, which are one of the four core components of the highly conserved SCF ubiquitin-ligase complex. Skp1 interacts directly with cullin and F-box proteins, with the latter functioning to selectively recruit substrate targets for ubiquitin ligation [23]. To address whether other *skr* genes or components of the SCF complex are also required for *gst-4* activation in response to juglone, we performed an RNAi screen against a sub-library of available *skr* dsRNA clones (*skr-3*, *5*, *7*, *8*, *9*, *10*, *11*, *12*, *13*, *15*, *17*, *18*, *19*, *20*, and *21*) and *cul-1*, which is the only cullin that is known to interact with SKR-1 and 2 [23,24]. Unlike *skr-1/2*, none of the other *skr* clones or *cul-1* had a significant effect on *Pgst-4*::*GFP* induction after juglone exposure (S7B Fig). Due to a general sickness and larval arrest of *cul-1*(*e1756*) null mutants [34], we were unable to confirm the *cul-1(RNAi)* results using a mutant. To enhance RNAi, we also tested *eri-1* RNAi hypersensitive worms fed *cul-1* dsRNA for two generations. Embryonic lethality (a well-established *cul-1* phenotype [35]) was observed in second generation worms fed *cul-1* dsRNA, but there was still no effect on *Pgst-4*::*GFP* when induced with juglone in second generation worms that were able to develop to the L4 stage (S7C and S7D Fig).

**Fig 6:**
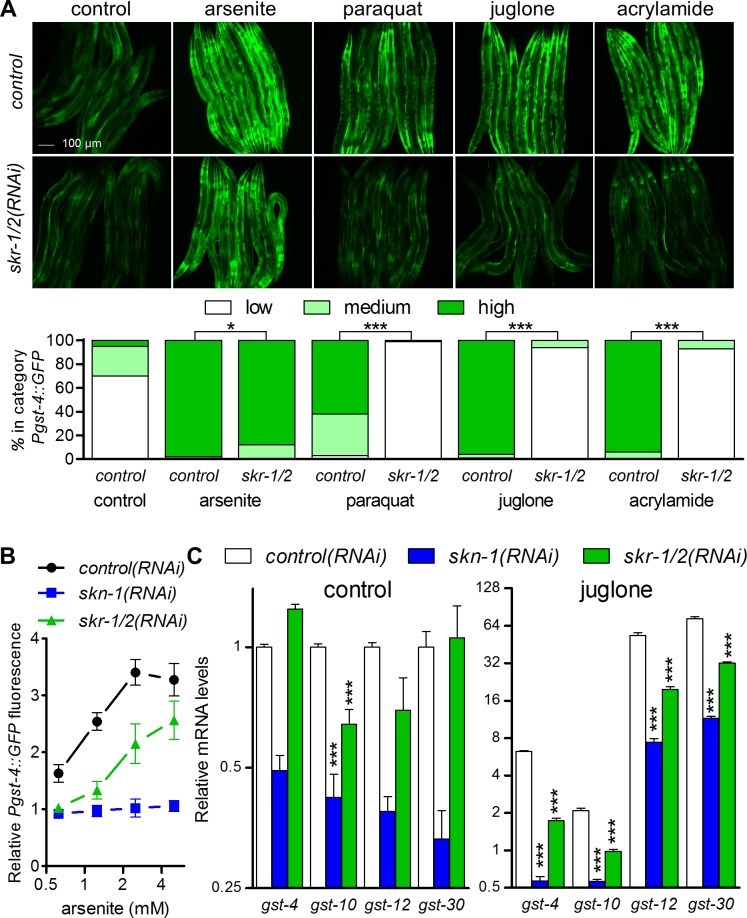
*skr-1/2* is required for induction of SKN-1 dependent detoxification genes. (A) *Pgst-4*::*GFP* fluorescence scoring and representative fluorescence micrographs of worms fed with control or *skr-1/2* dsRNA after exposure to 5 mM sodium arsenite for 1 h (recovered for 3 h on NGM agar to induce GFP), 35 mM paraquat for 2 h (recovered for 2 h), 38 μM juglone for 3 h (recovered for 1 h), or 7 mM acrylamide for 4 h (no recovery). *n* = 70–89 worms from 3 independent trials, *P<0.05, ***P<0.001 as determined by Chi-Square test. (B) Relative *Pgst-4*::*GFP* fluorescence measured by a plate reader after a 6 h exposure to a range of arsenite concentrations; all values are normalized to *control(RNAi)* with no arsenite. *n* = 4–8 wells of worms in a 384 well plate; P<0.001 for *skr-1/2(RNAi)* and *skn-1(RNAi)* versus *control(RNAi)* at all concentrations except for the lowest. (C) Fold changes in mRNA of *gst-4*, *gst-10*, *gst-12*, and *gst-30* relative to control (no stressors) in worms with control, *skn-1*, or *skr-1/2(RNAi)* after exposure to 38 μM juglone for 3 h. mRNA levels were normalized to *rpl-2;* values are means plus standard error of *n* = 4 replicates of 200–400 worms. All genes were induced significantly by juglone (P<0.001); ***P<0.001 compared to *control (RNAi)*.

To investigate the role of *skr-1/2* in response to reactive compounds, we tested its requirement for *Pgst-4*::*GFP* induction during exposure to arsenite, paraquat, juglone, and acrylamide. *skr-1/2(RNAi)* strongly inhibited induction of *Pgst-4*::*GFP* with juglone, paraquat, and acrylamide and had a smaller effect with arsenite (Fig 6A); a role for *skr-1/2* in *Pgst-4*::*GFP* induction by arsenite was confirmed in a more sensitive plate reading assay at four doses (Fig 6B). The requirement for *skr-1/2* was also tested with qPCR of four *gst* mRNAs directly controlled by SKN-1 under control conditions and after exposure to 38 μM juglone for 3 h (Fig 6C). Loss of *skr-1/2* only reduced expression of one of the *gst* genes under control conditions. Juglone activated all four *gst* mRNAs (P<0.001, Fig 6C), and as expected, *skn-1(RNAi)* strongly reduced induction of all four *gst* genes in juglone. Silencing of *skr-1/2* also strongly inhibited induction of all four *gst* genes by juglone by 57–74%. Therefore, *skr-1/2* is partially required for induction of core SKN-1 dependent detoxification genes by diverse reactive compounds.

### Global RNA-seq analysis demonstrates that *skr-1/2* is specifically required for *skn-1* transcriptional responses

**Fig 7:**
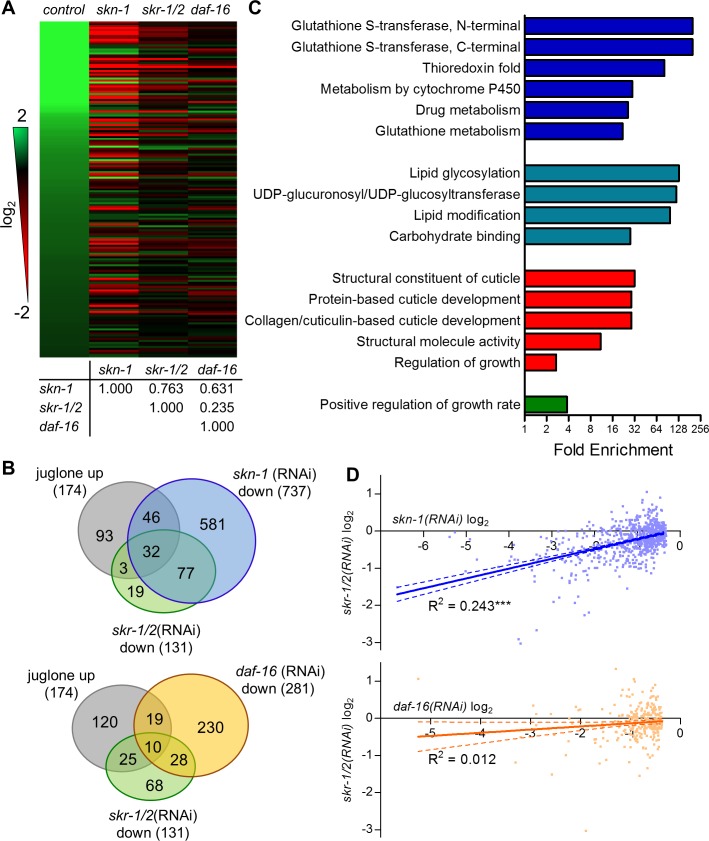
Whole transcriptome profiling reveals correlation between loss of *skn-1* and *skr-1/2*. (A) Heat map of log_2_ fold-change of 174 genes found to be significantly up-regulated by juglone (38 μM for 3 h) with their corresponding fold-changes in worms fed with *skn-1*, *skr-1/2*, or *daf-16* dsRNA compared to control dsRNA. Fold-change analysis for RNA-seq data were carried out using the CuffDiff application in Galaxy with q-value <0.05 determined by a false discovery rate of <5%. *n =* 3 replicates of 1,000–2,000 worms. Coefficients of correlation are listed below the map and 95% confidence intervals are as follows: *skn-1* and *skr-1/2* (0.693–0.818), *skn-1* and *daf-16* (0.533–0.712), and *skr-1/2* and *daf-16* (0.090–0.370). (B) Venn diagrams showing numbers of genes overlapping. (C) DAVID functional enrichment analysis of *skr-1/2(RNAi)* down-regulated genes. (D) Linear regression analysis of all genes down-regulated by either *skn-1* or *daf-16(RNAi)* plotted against their fold change with *skr-1/2(RNAi)*. ***P<0.001 as determined by linear regression F-test.

To obtain a global view of gene regulation by *skr-1/2*, we extracted RNA from worms grown on control, *skr-1/2*, and *skn-1(RNAi)* after exposure to juglone and identified differentially expressed genes (DEG) using unbiased whole-genome RNA sequencing (RNA-seq). The FOXO transcription factor *daf-16* is also widely involved in detoxification responses [36] and a previous study implicated *skr-1/2* in regulation of a DAF-16 dependent gene and in promoting longevity in *daf-2* mutant worms, which have elevated DAF-16 activity [33]. Therefore, we also included *daf-16(RNAi)* to evaluate the specificity of gene regulation by *skr-1/2*. We identified 309 (174 up, 135 down) DEG by juglone, 273 (142 up, 131 down) DEG by *skr-1/2(RNAi)*, 1241 (517 up, 737 down) DEG by *skn-1(RNAi)*, and 562 (281 up, 281 down) DEG by *daf-16(RNAi)* (Fig 7A and 7B, S8A and S8B Fig and S1 Table).

A heat map of all genes up-regulated by juglone is shown in Fig 7A demonstrating that many of the strongest up-regulated genes were dependent on both *skn-1* and *skr-1/2*. Correlation coefficients are shown below the heat map for all comparisons demonstrating a much higher correlation of *skr-1/2* with *skn-1* (0.763) than with *daf-16* (0.235) for the genes up-regulated by juglone. Of the 174 genes up-regulated by juglone, 78 were *skn-1* dependent and 35 were *skr-1/2* dependent (Fig 7B). Of the 35 *skr-1/2* dependent juglone induced genes, almost all (32, or 91%) were also *skn-1* dependent. Furthermore, 110 of the 131 (84%) total *skr-1/2* dependent genes were also *skn-1* dependent; 14.7% of all *skn-1* dependent genes were *skr-1/2* dependent. Compared to *skn-1(RNAi)*, *daf-16(RNAi)* affected fewer overall genes but had a similar proportional overlap with *skr-1/2* (13.5%, Fig 7B). Using DAVID analysis, *skr-1/2* dependent genes were primarily enriched for glutathione mediated detoxification and metabolism, glucosyltransferase activities, and collagen/cuticle development (Fig 7C), while the genes up-regulated by *skr-1/2(RNAi)* were enriched for regulation of growth rate, collagen, and metal ion binding (S8C Fig). Genes up-regulated by *skr-1/2(RNAi)* had little overlap with genes influenced by juglone (S8B Fig).

To gain deeper insights into the gene expression effects of *skr-1/2* relative to *skn-1* and *daf-16*, we performed linear regression analysis on all genes that were down-regulated by *skn-1(RNAi)* or *daf-16(RNAi)*, regardless of how they were affected by juglone, by plotting fold change with *skr-1/2(RNAi)* versus fold change by *skn-1* or *daf-16(RNAi)* (Fig 7D). Fold-changes for *skr-1/2(RNAi)* were significantly correlated with fold-changes for all genes significantly downregulated by *skn-1(RNAi)* (slope = 0.265±0.02, R^2^ = 0.24, P < 0.0001) (Fig 7D). No correlation was observed between *skr-1/2* and *daf-16(RNAi)* for all genes significantly downregulated by *daf-16(RNAi)* (slope = 0.08±0.05, R^2^ = 0.01, P = 0.07) (Fig 7D). A significant, but very small, correlation was found between *skr-1/*2 and *skn-1(RNAi)* for genes up-regulated by *skn-1(RNAi)*, not for genes up-regulated by *daf-16(RNAi)* (S8D Fig). Taken together, these results reveal that *skr-1/2* is required for expression of a subset of detoxification and extracellular matrix genes that are largely (84%) nested within a set of *skn-1* dependent genes and not correlated with *daf-16* dependent genes.

### Loss of *skr-1/2* reduces oxidative stress resistance

**Fig 8:**
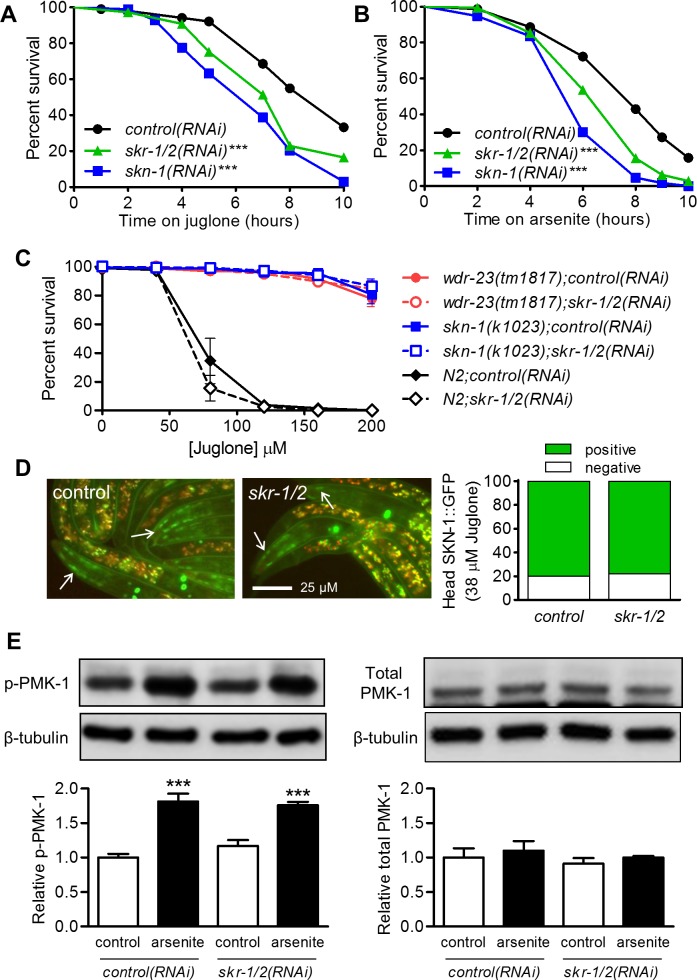
*skr-1/2* is required for juglone and arsenite resistance but not for SKN-1 accumulation or PMK-1 phosphorylation. (A) Survival of *eri-1* worms fed control, *skn-1*, or *skr-1/2* RNAi on 125 μM juglone after a 2 h pre-treatment at 38 μM. ***P<0.001 compared to control RNAi as determined by Log-rank (Mantel-Cox) test. *n* = 98–109 worms from a single trial; statistics and results from two other trials are shown in S9 Fig. (B) Survival of N2 worms fed control, *skn-1*, or *skr-1/2* RNAi on 10 mM arsenite. ***P<0.001 compared to control RNAi as determined by Log-rank (Mantel-Cox). *n* = 140–170 worms from a single trial; statistics and results from two other trials are shown in S9 Fig. (C) Survival of worms with enhanced SKN-1 activity exposed to a range of juglone concentrations for 16 h. *skr-1/2(RNAi)* did not significantly decrease survival at any concentration in any strain. *n* = 4 independent trials of 12–78 worms per condition and trial. (D) Animals with integrated SKN-1b/c::GFP were fed with either control or *skr-1/2(RNAi)* and treated with 38 μM juglone for 3 h and accumulation of head SKN-1b/c::GFP was scored. Representative fluorescence micrographs of head SKN-1b/c::GFP are shown with arrows marking GFP. *n =* 45 to 54 worms. (E) Phosphorylation of PMK-1 was measured in worms fed control or *skr-1/2* dsRNA and exposed to 5 mM sodium arsenite for 1 h. PMK-1 phosphorylation levels were normalized to β-tubulin and then to control (without stressor); β-tubulin was detected on the same blots after stripping. Total PMK-1 was measured on the same lysates in a different blot and also normalized to β-tubulin. Values are mean plus standard error for densitometry of protein bands from *n =* 3 replicates of >1,000 worms. Representative immunoblot bands are shown, ***P<0.001 relative to control. There was no significant effect of *skr-1/2(RNAi)*.

We next examined the requirement of *skr-1/2* for juglone and arsenite resistance. In our hands, *skn-1(RNAi)* often does not have a reproducible effect on survival of juglone in worms that have not previously been exposed suggesting that basal activity may not play a large role in survival of an acute lethal juglone dose. We then pre-conditioned worms to a low and hormetic level of juglone (38 μM for 2 h), which strongly activates SKN-1 dependent genes (Fig 2B) and dramatically increases resistance [37], before measuring survival in a much higher lethal dose (125 μM). In these experiments, *skn-1(RNAi)* significantly decreased juglone survival compared to control worms in all three trials and *skr-1/2(RNAi)* significantly decreased survival in two of three trials (Fig 8A and S9 Fig). In 10 mM arsenite, either *skn-1(RNAi)* or *skr-1/2(RNAi)* significantly decreased survival compared to the control worms (Fig 8B and S9 Fig). These results show that *skr-1/2* is required for oxidative stress resistance.

We next tested the effects of *skr-1/2(RNAi)* on juglone survival in two strains with greatly increased SKN-1 activity, *wdr-23*(*tm1817*) loss of function and *skn-1*(*k1023*) gain of function [10]. As expected, both of these strains were highly resistant over a range of juglone concentrations compared to N2, and *skr-1/2(RNAi)* did not have a reproducible effect on juglone resistance in N2 worms not previously exposed to juglone (Fig 8C). As expected given that *skr-1/2* appears to regulate *gst-4* upstream from *wdr-23* (Fig 5B), *skr-1/2(RNAi)* did not reduce resistance of either *wdr-23*(*tm1817*) or *skn-1*(*k1023*) worms (Fig 8C).

### *skr-1/2(RNAi)* does not affect nuclear accumulation of SKN-1::GFP or PMK-1 phosphorylation

Having established *skr-1/2* as a requirement for SKN-1 dependent detoxification responses, we conducted additional experiments to gain insight into potential mechanisms. We demonstrated above that *skr-1/2* likely regulates detoxification gene expression upstream from WDR-23 and SKN-1 (Fig 5B). To test if *skr-1/2* is required for nuclear localization of SKN-1, we performed *skr-1/2(RNAi)* in a strain carrying the SKN-1b/c::GFP transgene and scored accumulation after exposure to juglone. Because we did not observe an increase of SKN-1b/c::GFP accumulation in the intestine nuclei by juglone (Fig 3A), we scored the effects of *skr-1/2(RNAi)* on SKN-1b/c::GFP accumulation in the head region. RNAi against *skr-1/2* failed to prevent accumulation of SKN-1b/c::GFP in the head after juglone exposure (Fig 8D).

We next determined if *skr-1/2* loss influences phosphorylation of PMK-1. RNAi against *skr-1/2* had no effect on total or phosphorylated PMK-1 levels under basal conditions or when increased by arsenite (Fig 8E), a condition that strongly increased phosphorylated PMK-1 levels (Fig 1). Taken together, these data suggest that although *skr-1/2* is required for activation of SKN-1 target genes by stress, it does so without observable changes to SKN-1 nuclear accumulation or p38 MAPK phosphorylation.

### An *skr-1* deletion mutant decreases SKN-1 target gene activation

As mentioned earlier, *skr-1* and *skr-2* are very similar even at the nucleotide level (83% identical). In order to investigate if one or both are required for *gst* induction, we conducted qPCR experiments with *skr-1*(*tm2391*) and *skr-2*(*ok1938*) deletion mutants. *skr-1*(*tm2391*) and *skr-2*(*ok1938*) homozygotes are each maternal effect embryonic lethal and *skr-1* mutants also display late larval arrest [34]; RNA was isolated by picking healthy homozygote larvae at the L3 and early L4 stages. The *skr-1*(*tm2391*) allele reduced mRNA levels of four juglone induced *gst* genes (S10A Fig). The *skr-2*(*ok1938*) allele only slightly reduced mRNA levels for two genes (*gst-4* and *12*) and increased induction of *gst-10* and *gst-30*. Interestingly, *skr-2*(*ok1938*) had a ~40% reduction in *skr-1* mRNA levels (S10B Fig), likely due to a partial deletion of the *skr-1* promoter in this mutant; however, this reduction in *skr-1* mRNA appeared to have minimal effect on the activation of *skn-1* dependent genes in response to juglone. These results help confirm a role of *skr-1* and are consistent with SKR-2 being less important than SKR-1 for SKN-1 pathway activation.

To test if overexpression of *skr-1/2* alone is sufficient to increase expression of SKN-1 transcriptional targets, we generated a transgenic worm overexpressing a 4 kb DNA fragment that contained full genomic sequences of both *skr-1* and *skr-2* and an integrated *Pgst-4*::*GFP* reporter. Transgenic worms carrying the 4 kb DNA fragment overexpressed (oe) both *skr-1* and *skr-2* mRNA, but had wild-type levels of *Pgst-4*::*GFP* fluorescence (S11A and S11B Fig); qPCR confirmed that the mRNA levels of SKN-1 transcriptional targets *gst-4* and *gcs-1* were unaffected by *skr-1/2* overexpression at basal conditions, and the overexpression also did not further activate SKN-1 target transcription during juglone exposure (S11C Fig). Therefore, overexpression of *skr-1/2* is not sufficient to activate the SKN-1 stress response.

### SKR-1 is expressed broadly and interacts with and influences WDR-23

**Fig 9:**
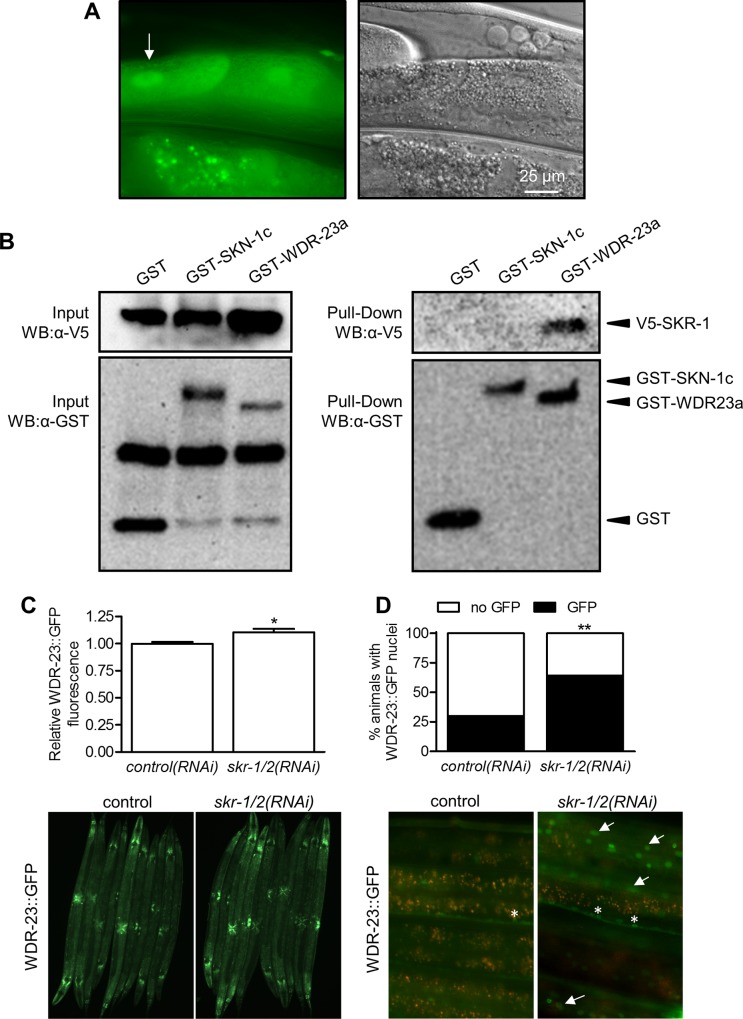
SKR-1 is expressed broadly and interacts with WDR-23. (A) Fluorescence and differential interference contrast micrographs showing cytoplasmic and nuclear (arrow) expression of SKR-1::GFP in the intestine (QV254). (B) SKR-1 interacts with WDR-23. HEK293 cells were co-transfected with V5-SKR-1 fusion protein along with either GST only vector (pDEST27), GST-SKN-1c fusion protein, or GST-WDR23a fusion protein. Complexes were captured with GSH beads and interactions with SKR-1 were determined by immunoblotting with anti-V5 mAb. Co-pulldown of V5-SKR-1 with GST-WDR-23a was also detected in a separate independent trial. (C-D) Total WDR-23::GFP fluorescence (normalized to RFP) and percentage of worms with visible nuclear WDR-23::GFP fluorescence with and without *skr-1/2(RNAi)*. *P<0.05 and **P<0.01. Arrows point to hypodermal nuclei and asterisks mark neuronal cells.

To determine the expression pattern of SKR-1, we generated transgenic worms expressing a SKR-1::GFP fusion protein. Consistent with a previous study [24], SKR-1::GFP was expressed throughout the worm, with SKR-1::GFP highly expressed in the intestine, pharynx, neurons, and spermatheca (S12 Fig); intracellular distribution of SKR-1 was not previously determined. Within the large cells of the intestine, we observed SKR-1::GFP in the cytosol and nuclei suggesting that the protein is broadly distributed within cells (Fig 9A). To test if SKR-1 is regulated under stress, we treated this transgenic worm to conditions that activate SKN-1. Exposure to juglone and arsenite did not result in obvious changes in expression or localization of SKR-1::GFP (S11D Fig).

To test whether SKR-1 might interact with key members of the SKN-1 pathway, we co-transfected either full length SKN-1c or WDR-23a fused to an N-terminal GST tag together with SKR-1 fused to an N-terminal V5 fusion tag in HEK293 cells and performed GST pull-downs. Pulldown of GST-SKN-1c failed to capture SKR-1 as determined by Western blot using a V5 monoclonal antibody (Fig 9B). Alternatively, SKR-1 was captured by pull-down of GST-WDR-23a. We also used a strain expressing an integrated transgene of *wdr-23a* cDNA fused to GFP [38], which rescues misexpression of a *gst-4* transgene in *wdr-23* mutants, to test if SKR-1/2 might regulate WDR-23 *in vivo*. As shown in Fig 9C, *skr-1/2(RNAi)* had a significant but very small effect on WDR-23::GFP fluorescence levels. Alternatively, *skr-1/2(RNAi)* doubled the proportion of worms with obvious nuclear localization in the hypodermis (Fig 9D). Obvious changes to intestinal WDR-23::GFP were not observed.

## Discussion

**Fig 10:**
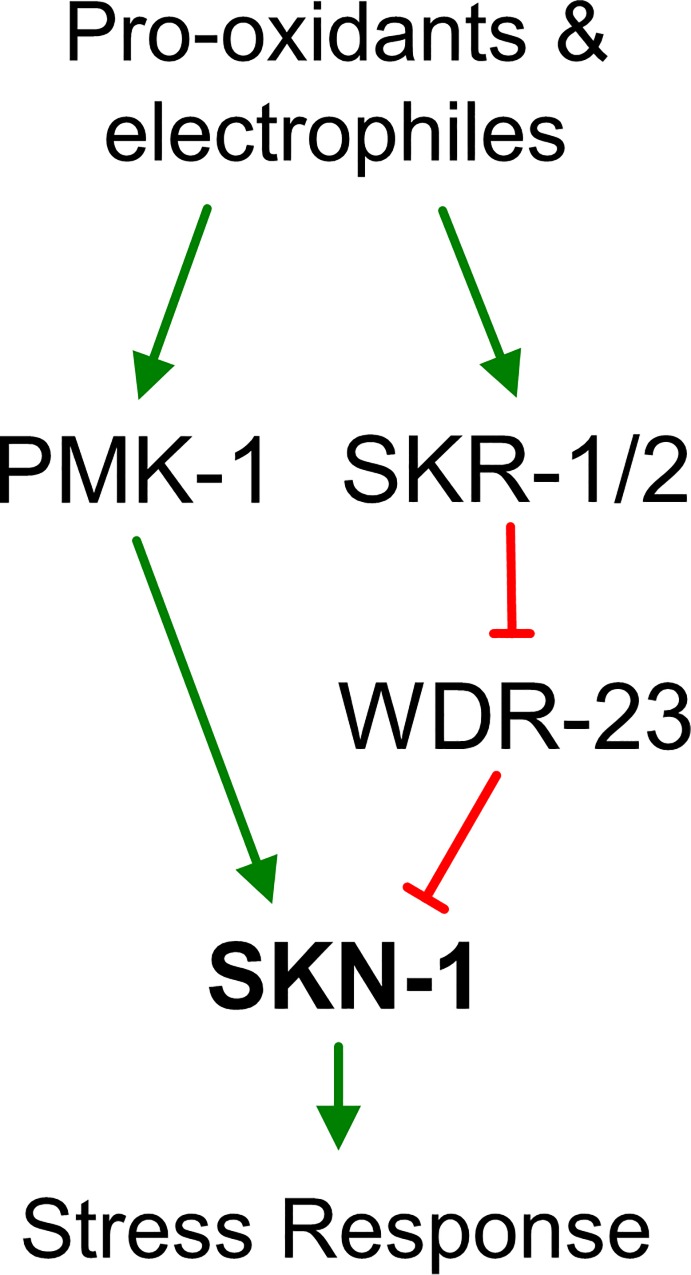
Model of SKR-1 activation by pro-oxidants and reactive small molecules. In response to pro-oxidants, PMK-1 is activated and contributes to SKN-1 activation. Additional parallel or compensatory mechanisms likely exist given the ability of detoxification genes to be induced strongly without PMK-1 (Fig 2B). In response to diverse pro-oxidants and electrophiles, SKN-1 dependent gene expression is induced by a mechanism that requires SKR-1/2 (Figs 5 and 6). SKR-1/2 is not required for phosphorylation of p38 MAPK (Fig 8) and likely functions independently. SKR-1 acts upstream of WDR-23 (Fig 5), is able to interact with WDR-23 (Fig 9), and influences its nuclear levels suggesting a mechanism through this direct SKN-1 repressor.

In *C*. *elegans*, the p38 MAPK signaling cascade activates SKN-1 in the intestine during oxidative stress induced by arsenite exposure [19] and during pathogen infection [20,21]. Here, we demonstrate striking variation in PMK-1 activation kinetics among diverse pro-oxidant and electrophilic compounds that all strongly induce SKN-1 dependent transcriptional responses. SKN-1 can also accumulate in the nuclei of tissues other than the intestine partially independent of PMK-1. To begin defining other regulatory pathways that may function parallel to PMK-1, we leveraged the genetic tractability of *C*. *elegans* to identify a novel role for the highly conserved Skp1 homologs *skr-1*/*2* in the SKN-1 mediated stress response. SKR-1 interacts with and influences the localization of the SKN-1 repressor WDR-23, and loss of *skr-1/2* inhibits the expression of *skn-1* dependent detoxification genes and impairs survival during exposure to pro-oxidants. A revised working model for SKN-1 regulation by reactive small molecules that incorporates our findings is presented in Fig 10 and discussed below.

### PMK-1 phosphorylation and intestinal SKN-1::GFP accumulation vary with different pro-oxidants that activate SKN-1 dependent stress responses

Phosphorylation of PMK-1 at activating residues by arsenite and pathogen exposure is associated with intestinal accumulation of nuclear SKN-1 consistent with the prevailing model in which PMK-1 phosphorylates SKN-1 in the intestine during oxidative stress and causes nuclear accumulation [19]. Interestingly, PMK-1 phosphorylation kinetics varied greatly between different sub-lethal pro-oxidant and electrophile exposures that all strongly activate SKN-1 dependent detoxification gene expression (Fig 1). Acrylamide even decreased PMK-1 phosphorylation levels during chronic exposure, but caused strong and sustained *Pgst-4*::*GFP* induction (Figs 1 and 2 and S3 Fig). We also observed strong SKN-1 dependent detoxification gene activation in *pmk-1* deletion mutants during sub-lethal exposure to arsenite and juglone (Fig 2) and SKN-1 nuclear accumulation in tissues other than the intestine (Fig 3 and S4 Fig) and in *pmk-1* deletion mutants (Fig 4).

Importantly, SKN-1 dependent gene induction was partially dependent on *pmk-1* (Fig 2B), which is consistent with a recent study showing that *sek-1* and *pmk-1* are required for induction of a downstream target of SKN-1 in neurons (*nlg-1*) that promotes survival of juglone [39]. We also cannot rule out the possibility that SKN-1 accumulates in the intestine with juglone at levels below what we are able to detect. Our data establish that pro-oxidants and electrophiles are capable of activating SKN-1 and its downstream detoxification genes outside the intestine and with varying degrees of SKN-1::GFP nuclear accumulation and PMK-1 activation suggesting the presence of parallel or compensatory mechanisms; e.g., other kinases downstream from NSY-1/SEK-1 (such as PMK-2 or PMK-3) or other unknown pathways. It is also possible that other transcription factors could help compensate.

### *C*. *elegans skr-1/2* is required for detoxification gene induction

SKR homologs function in SCF multi-subunit E3 ubiquitin ligases that are conserved from yeast to humans. In yeast and humans, a single SKR named Skp1 forms the SCF complex with Rbx, Cul1, and various F-box proteins to promote protein ubiquitination [40,41]. Skp1 also functions as a scaffold in protein complexes independently of SCF [42,43,44,45]. In *C*. *elegans*, 21 *skr* genes have been identified [23,24]. Loss of function of either *skr-1* or *2* results in embryonic arrest that is characterized by excessive cell numbers and hyperplasia [23,24]. Interestingly, *skr-1/2(RNAi)* delays degradation of SKN-1 during embryonic development leading to a delay in development [46]. SKR-1/2 was previously shown to be required for longevity in *daf-2* mutants and not wildtype worms [33], but a role in stress responses has not been reported. In this study, we identified a new role for *skr-1/2* in permitting *skn-1* dependent stress responses in larval and adult stages of *C*. *elegans*.

*skr-1/2* were required for transcriptional induction of a SKN-1 dependent *gst-4* reporter by diverse pro-oxidants at sub-lethal doses (Fig 6) and for resistance to lethal doses of juglone and arsenite (Fig 8A and 8B). We found that *skr-1/2(RNAi)* did not reduce levels of total or phosphorylated PMK-1 (Fig 8E), and in our RNA-seq data, knockdown of *skr-1/2* did not alter mRNA levels of core MAPK genes (*nsy-1*, *sek-1*, or *pmk-1*) (S1 Table). Although it remains possible that *skr-1/2* could affect protein levels of NSY-1 or SEK-1, these data raise the possibility of SKR-1/2 functioning by a different mechanism.

### *skr-1/2* specifically regulates *skn-1* dependent responses

The function of *skr-1/2* in our study was shown to be tightly linked to SKN-1. Knockdown of *skr-1/2* did not block heat-shock or osmotic stress transcriptional response reporters (S6 Fig) and *skr-1/2* dependent genes were enriched for functions in detoxification and cuticle collagen (Fig 7), which are both also enriched among *skn-1* dependent genes [12,30,47]. Furthermore, our RNA-seq data demonstrated that a strikingly large majority (84%) of *skr-1/2* dependent genes were also *skn-1* dependent and that expression changes caused by *skr-1/2* and *skn-1* RNAi were correlated (Fig 7). *skr-1/2* had no correlation with genome-wide *daf-16* dependent expression (Fig 7).

### How might SKR-1/2 regulate SKN-1 activity?

The SCF ubiquitin ligase complex has previously been reported to negatively regulate Nrf2 protein levels in human cells; phosphorylation of Nrf2 by glycogen synthase kinase 3 promotes ubiquitin mediated Nrf2 degradation by the SCF complex *via* its interaction with the β-transducin repeat-containing protein (β-TrCP) F-box [48]. Our results show that *skr-1/2* Skp1 homologs in *C*. *elegans* positively regulate *skn-1* mediated stress responses. RNAi against the gene encoding the central scaffold of the *C*. *elegans* SCF complex, *cul-1*, failed to mimic the effects of *skr-1/2(RNAi)* on regulation of the *gst-4* promoter during exposure to juglone (S7 Fig). However, we cannot rule out a role for *cul-1* because of the possibility of residual CUL-1 function after RNAi treatment.

*C*. *elegans* and *Drosophila melanogaster* each contain expanded families of Skp1 homologs whereas yeast and vertebrates, including humans, have only one member [23,24]. Having multiple Skp1 homologs has been hypothesized to permit the evolution of more flexible and variable functions. Even in yeast and human cells where single homologs are present, evidence has been provided for Skp1 functioning independently of the SCF complex to regulate membrane protein recycling, kinetochore function, ion transport, and protein degradation [42,43,44,45,49]. In all these cases, the molecular role of Skp1 appears to be as a scaffold to assist in protein complex assembly.

In *C*. *elegans*, SKN-1 is regulated directly by WDR-23 and PMK-1 [16,19]. Our data do not support a positive association between SKR-1/2 and PMK-1 function as *skr-1/2(RNAi)* did not reduce PMK-1 protein levels or its stress activated phosphorylation (Fig 8E). Instead, SKR-1 has the potential to interact with WDR-23 and the requirement of *skr-1/2* for *skn-1* dependent gene induction was abolished in a *wdr-23* null mutant (Fig 5B). WDR-23 is a direct repressor of SKN-1 that is present in many cells [16,25,50], and loss of *wdr-23* promotes stress resistance and a long-lived phenotype that is *skn-1* dependent [10]. SKR-1 was present throughout cells including within some nuclei (Fig 9A) where it could interact with WDR-23. Our genetic interaction and WDR-23::GFP results (Figs 5B, 9C and 9D) are consistent with SKR-1/2 functioning to negatively regulate WDR-23 accumulation in nuclei likely by serving as a scaffold to influence protein complex assembly (Fig 10). SKN-1 dependent genes are extremely sensitive to changes in WDR-23 function [16] and one possible mechanism is for SKR-1/2 to negatively regulate WDR-23 protein levels *via* ubiquitination. In this model, loss of *skr-1/2* would increase WDR-23 levels and repression of SKN-1.

Importantly, we did not observe accumulation of either SKN-1b/c or SKN-1op(a/b/c) reporters in the intestine after juglone exposure and *skr-1/2* RNAi did not impair SKN-1b/c::GFP accumulation in head nuclei (Fig 8D). Although SKR-1/2 could influence SKN-1 accumulation at levels that we were not able to detect, it is also possible that SKR-1/2 and WDR-23 may be able to regulate SKN-1 by alternative mechanisms such as DNA binding or transactivation activity. Future experiments to investigate these alternatives and to identify other SKR-1 and WDR-23 interacting proteins required for the detoxification response could help clarify the molecular mechanisms of WDR-23 and SKN-1 regulation. Induction of SKN-1 detoxification genes can also be decoupled from nuclear accumulation in the intestine during genetic impairment of translation and fatty acid metabolism pathways [51,52]. It remains to be seen if SKR-1/2 plays a role under these conditions.

### Summary

Our study provides evidence that PMK-1 phosphorylation and SKN-1 nuclear localization are differentially regulated in response to different reactive small molecule exposures that activate SKN-1 dependent detoxification responses. We also identify SKR-1/2 as required for SKN-1 dependent detoxification gene induction in response to diverse pro-oxidants and electrophiles that may interact with and regulate WDR-23. SKN-1 is activated by a broad range of different conditions and our study highlights the fact that distinct upstream regulatory pathways are present that may permit tailored responses to oxidative and reactive small molecule stressors. These findings and the identification of SKR-1/2 as a key regulator lay the foundation for defining a novel SKN-1 regulatory mechanism that involves WDR-23.

## Materials And Methods

### *C*. *elegans* strains

*C*. *elegans* strains were grown and maintained at 20°C using standard methods [53], unless noted otherwise. The following strains were used: wild-type N2 Bristol, VP596 *dvIs19* [*pAF15*(*Pgst-4*::*GFP*::*NLS*)]*; vsIs33*[*Pdop-3*::*RFP*]), QV65 *gpIs* [*Phsp-16*.*2*::*GFP*]; *vsIs33*, CF1580 *daf-2*(*e1370*) *III; muIs84* [(*pAD76*) *sod-3p*::*GFP + rol-6*], QV25 *wdr-23*(*tm1817*)*; eri-1*(*mg666*)*IV; dvIs19*, QV130 *skn-1*(*k1023*)*; dvIs19*, KU25 *pmk-1*(*km25*)*IV*, KU4 *sek-1*(*km4*)*X*, LD1 *ldIs7* [*skn-1B/C*::*GFP + pRF4*(*rol-6*(*su1006*))], LD1008 [*skn-1(operon)*::*GFP*, rol-69su1006)], VP537 *eri-1*(*mg666*)*IV; dvIs19*, GR1373 *eri-1*(*mg366*)*IV*, VP604 *kbIs24* [*Pgpdh-1*::*dsRed2;Pmyo-2*::*GFP;unc-119*] X, QV254 *zjEx114* [*SKR-1*::*GFP; Pmyo-2*::*tdTomato*], QV256 *zjEx115* [*skr-1/2 gDNA; Pmyo-2*::*tdTomato; Pmyo-3*::*dsRed*], QV288 *pmk-1*(*km25*)*IV*; *ldIs7*, VC1439 *skr-2*(*ok1938*) *I/hT2* [*bli-4*(*e937*) *let-*?(*q782*) *qIs48*] (*I;III*), CU6110 *skr-1*(*tm2391*) *I/hT2 I;III; +/hT2 I;II*, and KHA116 *unc-119(ed3); chuIs116* [*wdr-23p*::*wdr-23a(cDNA)*::*GFP*, *unc-119*].

### RNAi and genome-wide RNAi screening

RNAi was performed as described previously [16] by feeding worms strains of *E*. *coli* [HT115(DE3)] that are engineered to transcribe double stranded RNA (dsRNA) homologous to a target gene. For screening, L1 larvae of VP537 worms were grown in liquid medium with dsRNA-producing bacteria for 3 days, and subsequently exposed to 38 μM juglone for 4 h and screened for *Pgst-4*::*GFP* expression with a Zeiss Stemi SV11 microscope. The entire ORFeome RNAi feeding library (Open Biosystems, Huntsville, AL) was screened with additional missing clones supplemented from the original genomic RNAi feeding library (Geneservice, Cambridge, United Kingdom). Clones that resulted in reduced *Pgst-4*::*GFP* were rescreened three additional times, clones with positive scores in all three trials were considered novel regulators of *gst-4* and subsequently sequenced for identification. With the exception of the genome-wide RNAi screen, all subsequent RNAi experiments were performed on nematode growth media (NGM) plates that were made with the addition of 50 μg mL^-1^ carbenicillin, 0.2% lactose, and seeded with appropriate HT115 RNAi bacteria and grown overnight before use. RNAi feeding was initiated at either synchronized L1 larvae stage, or at L4 to young adult stage for RNAi clones that displayed developmental effects when fed from L1. Bacteria with plasmid pPD129.36 were used as a control for non-specific RNAi effects. This control plasmid expresses 202 bases of dsRNA that are not homologous to any predicted *C*. *elegans* gene.

### Fluorescence microscopy with GFP reporters

To visualize SKN-1b/c::GFP, LD1 worms at L1/L2 stages were incubated with 5 mM sodium arsenite for 1 h, 5 mM sodium azide for 5 min, 35 mM paraquat for 2h, 38 μM juglone for 5–15 min, 1, 3, and 5 h, or 7 mM acrylamide for 5–15 min, 1, 4, 12, or 24 h. Worms were then washed and anesthetized with 5 mM levamisole. Anesthetized worms were mounted on 2% agarose pads and visualized and imaged using an Olympus BX60 microscope and Zeiss AxioCam MRm camera. Differential interference contrast and fluorescent images of SKN-1b/c:GFP and SKN-1op::GFP were taken. Both grayscale images taken by the GFP and RFP channels were merged into green and red channels respectively to produce a composite image using ImageJ (NIH). When needed for clarity, brightness and contrast adjustments were made equally to images from the same color channel and within the same experiment. Accumulation of SKN-1b/c::GFP and SKN-1op::GFP expression in intestinal nuclei was scored as in previous studies [19], while accumulation of SKN-1b/c::GFP and SKN-1op::GFP in the head regions were scored as either negative, referring to no observation of GFP signals in the head region, or positive, referring to GFP signals observed throughout the head region.

To visualize *Pgst-4*::*GFP*, young adult worms were treated with 5 mM sodium arsenite for 1 h in liquid NGM (and were washed 3x with NGM buffer and allowed to recover for 3 h on NGM agar), 35 mM paraquat for 2 h in liquid NGM (recovery for 2 h), 38 μM juglone in liquid NGM for 3 h (recovery for 1 h), or 7 mM acrylamide in liquid NGM for 4 h (no recovery). These conditions were all sub-lethal (S1A Fig) but strongly induce *Pgst-4*::*GFP* fluorescence (Fig 5A). For *Pgst-4*::*GFP* scoring, low refers to little to no GFP observed throughout the worm, medium refers to GFP signals observed only at the anterior and posterior ends of the worm, and high refers to GFP signals observed throughout the body. To visualize *Phsp-16*.*2*::*GFP*, young adult worms were exposed to 33°C for 1 h followed by 5 h recovery at 20°C; for *Pgpdh-1*::*RFP*, young adult worms were exposed to 250 mM NaCl for 24 h. To visualize effects of oxidants on SKR-1::GFP expression and localization, young adult worms were exposed to 38 μM of juglone for 3 h or 5 mM arsenite for 1 h.

### Real-time PCR

Quantitative real-time RT-PCR was used to measure mRNA levels in L4 to young adult stage worms fed with appropriate RNAi as described previously [16]. Worms were incubated with 5 mM sodium arsenite or 38 μM juglone in liquid NGM for 1 or 3 h at 20°C with gentle shaking. Total RNA from 200–300 worms was isolated with a Quick-RNA MicroPrep Kit (Zymo Research, Irvine, USA), and cDNA was synthesized using 1 μg of RNA with GoTaq 2-Step RT-qPCR System (Promega, Madison, WI, USA) following the manufacturer’s protocol. Quantitative real-time PCR was performed in 10 μL reactions in a Realplex ep gradient S Mastercycler (Eppendorf AG, Hamburg, Germany) with GoTaq Green Master Mix (Promega, Madison, WI) according to the manufacturer’s protocol. Data was analyzed by the standard curve method, with the housekeeping genes *rpl-2* and *cdc-42* used as internal reference controls. Primer sequences are available upon request.

### Western blotting

Synchronized L4 to young adult stage N2 worms were treated with compounds in liquid NGM while gently shaking at 20°C. Each treatment was accompanied by a control group of worms incubated in NGM buffer for the same duration. After incubation with each stressor, worms were washed three times with NGM buffer, and approximately 1,000 L4 to young adult stage worms were lysed in homogenization buffer for each replicate (homogenization buffer content: 50 mM Tris Base pH 7.5, 150 mM NaCl, 0.1% SDS, 0.5% NaDeoxycholate, 1x Halt™ protease inhibitor cocktail, and 1x Halt phosphatase inhibitor cocktail (LifeTechnologies, Cat# 78430, #78420, Rockford, IL). Worms were sonicated for complete lysis by ultrasonication (Misonix XL 2000, Farmingdale, NY). Worm lysates were centrifuged at 13,000 *x g* for 10 min at 4°C and supernatants were normalized for protein concentration with BCA protein assay (Pierce, Cat#23227) and collected for SDS-PAGE electrophoresis. Equal volume of lysates totaling 30 μg of proteins were loaded and separated by SDS-PAGE, and detected by immunoblotting with phosphorylated PMK-1 (1:2000; Promega, Cat# V1211x), total PMK-1 antibody (1:1000; gift from K. Matsumoto [54]), and β-tubulin antibody (1:100; Developmental Studies Hybridoma Bank, Cat # E7) with methods as previously described [16].

### Co-transfection and GST pulldown

HEK293 cells were cultured in Dulbecco modified Eagle medium supplemented with 10% fetal bovine serum, 4.5 g/l glucose, 584 mg/l L-glutamine, 100 mg/l sodium pyruvate, and 1 U/ml penicillin. Co-transfections were performed with Lipofectamine LTX with PLUS reagents (Life Technologies, Cat#1533810) using full length SKN-1c or WDR-23a cloned into pDEST27 vector and full length SKR-1 cloned into V5-DEST vector. Two days after transfection, cells were lysed with immunoprecipitation lysis buffer (Life Technologies, Cat # 87787) and pulldown was conducted using glutathione-Sepharose 4B at room temperature for 1 h (GE Healthcare, Cat#17-0756-01 Little Chalfont, United Kingdom). Beads from pulldowns were washed extensively with PBS+0.1% Triton-X and eluted with an equal volume of 2X SDS loading buffer by heating at 90°C for 5 minutes. Western blots were carried out as described above, with mouse anti-GST MAb (1:1,000; Santa Cruz Biotech, Cat #B-14 Dallas, TX), and mouse anti-V5 tag MAb (1:1000; Invitrogen, Cat #R960-25).

### Stress resistance assays

Juglone stress resistance assays were performed on synchronized worms at L4 stages fed HT115 bacteria. Worms were pretreated with 38 μM of juglone in liquid NGM for 2 h and subsequently transferred to 125 μM juglone for survival analysis similar to [37]. In the arsenite stress resistance assay, worms were incubated with 10 mM of arsenite in liquid NGM and analyzed for survival. Worms were considered dead if they did not display any movement in response to prodding with a thin wire. A total of three independent trials were performed for each survival assay.

### Whole transcriptome RNA sequencing

N2 worms were synchronized *via* by hypochlorite treatment and grown on RNAi for two days. Worms were then incubated in either NGM buffer (control) or 38 μM juglone for 3 h. RNA was extracted from ~1,000–2,000 worms per sample using the RNAqueous-Micro Total RNA Isolation Kit (ThermoFisher Scientific, Cat#AM1931). Total RNA was sent to The Yale Center for Genome Analysis for 75 nucleotide single-end sequencing in an Illumina HiSeq 2000. Raw sequencing data was processed using the public Galaxy server and mapped to the *C*. *elegans* genome (ce10). Using the Cufflink package and CuffDiff application from Galaxy [55], FPKM (Fragments Per Kilobase of transcript per Million mapped reads) were calculated and tested for differential expression with a FDR score of 5%. Differentially expressed genes were clustered for GO (Gene Ontology) analysis by DAVID for gene functional classification [56].

### Statistical analyses

Statistical significance was determined using Student’s T-test when two means were compared and a one-way analysis of variance (ANOVA) with Tukey’s or Bonferonni multiple comparison tests when three or more means were compared. Log-Rank tests in the OASIS online tool were used when survival curves were compared [57]. Chi-square tests were used to evaluate categorical data, and linear regression was used for testing correlations in gene expression. *P* values of <0.05 were taken to indicate statistical significance except for comparing more than two survival curves, in which Bonferonni adjustments were made to *P* values to account for repeated comparisons. Statistical significance is indicated in figures as *P<0.05, **P<0.01, ***P<0.001, and ns = not significant. Data is available at: https://figshare.com/s/fb874c6dc1aa9b77375c

## Supporting Information

S1 Table RNA sequencing results worms treated with juglone and dsRNA for *skn-1*, *skr-1/2*, and *daf-16*. Fragments Per Kilobase of transcript per Million mapped reads (FPKM) values are provided for triplicates.
(XLSX)

S1 Fig Doses of pro-oxidants used to activate SKN-1 are non-lethal. Percentage survival of worms after treatment with each oxidant were scored after 24 h recovery on NGM agar plate seeded with OP50 *E*. *coli*. *n =* 3 trials of 274–402 worms total.
(TIF)

S2 Fig Short-term exposure to arsenite, juglone, or acrylamide did not increase SKN-1b/c::GFP localization (top). Accumulation of intestinal and head SKN-1b/c::GFP after 5 or 15 min exposure to 2% azide, 5 mM arsenite, 38 μM juglone or 7 mM acrylamide, *n =* 63–84 worms per condition. ** P<0.01, ***P<0.001 from corresponding controls as determined by the Chi-Square test (bottom). Representative images of SKN-1b/c::GFP. Arrows mark head GFP and arrowheads mark intestinal nuclei.
(TIF)

S3 Fig Representative fluorescent micrographs and DIC images of worms expressing *Pgst-4*::*GFP* in the hypodermis and intestine after exposure to 7 mM acrylamide for 4 h or 38 μM juglone for 3 h and recovered for 1 h. Arrows mark *Pgst-4*::*GFP* in hypodermal nuclei, arrowheads mark *Pgst-4*::*GFP* in the intestine.
(TIF)

S4 Fig SKN-1b/c::GFP localizes to multiple tissues during stress. (A) Representative fluorescence micrographs of worms expressing SKN-1b/c::GFP treated with NGM buffer (control) or 5 mM sodium arsenite for 1h fed with control or *skn-1(RNAi)*. (B) Scoring of intestinal and head SKN-1op::GFP and representative fluorescence micrographs. *n* = 52–71 worms. ***P<0.001 from corresponding controls as determined by Chi-Square tests. Scoring is the same as in Fig 2. (C) Representative fluorescence micrographs and differential interference contrast (DIC) images showing worms expressing SKN-1b/c::GFP in the hypodermis after exposure to 5 mM sodium arsenite for 1 h. Arrows mark head GFP and arrowheads mark ASI neurons.
(TIF)

S5 Fig SKN-1 in the hypodermis and intestine contribute to survival of juglone. Wildtype and tissue-specific RNAi worms fed with control and *skn-1(RNAi)* were exposed to 38 μM juglone for 2 h and then the concentration was raised to a total of 125 μM and survival was measured for up to 10 h. Graphs for trial 2 are shown above and a summary of the results for all three trials is show below. *skn-1(RNAi)* reduced survival in all three strains.
(TIF)

S6 Fig Novel regulators of *Pgst-4*::*GFP* are not required for core heat shock or osmotic stress responses. (A-B) Animals integrated with *Phsp16*.*2*::*GFP* or *Pgpdh-1*::*RFP* were fed bacteria producing dsRNA and exposed to either heat shock (transfer from 20 to 33°C for 1 h and 5 h recovery for *Phsp16*.*2*::*GFP*) or osmotic stress (transfer from 51 to 250 mM NaCl for 24 h for *Pgpdh-1*::*RFP*). Fluorescence was scored as in Fig 4. ***P<0.001 compared to control as determined by Chi-Square tests.
(TIF)

S7 Fig RNAi of other *skr* genes and *cul-1* does not inhibit *Pgst-4*::*GFP* induction. (A) *skr-1* and *skr-2* RNAi have similar effects on *Pgst-4*::*GFP* induced by juglone. (B) RNAi screen against a sub-library of genes functioning within the SCF complex in animals carrying the *Pgst-4*::*GFP* reporter after juglone exposure (38 μM for 3 h). ***P<0.001 compared to control as determined by Chi-Square tests. (C) *Pgst-4*::*GFP* scoring and representative fluorescence micrographs of *eri-1* worms fed with control or *cul-1(RNAi)* for two generations and exposed to 38 μM juglone for 3 h. (A-C) *n =* 53–139 worms. (D) High penetrance of embryonic lethal and larval arrest phenotypes are observed in F2 generation of *cul-1* RNAi fed *eri-1* worms. F1 mothers were allowed to lay eggs and then removed for 24 h before taking an image; note a high number of dead eggs and sick L1 larvae.
(TIF)

S8 Fig Whole genome analysis of genes up-regulated by RNAi of *skn-1*, *skr-1/2*, or *daf-16*. (A) Heat map of fold-changes in 135 genes down-regulated by juglone with their corresponding fold-changes in worms fed with dsRNA for *skn-1*, *skr-1/2* or *daf-16*. *n =* 3 replicates of 1,000–2,000 worms. Coefficients of correlation are listed below the heat map and 95% confidence intervals are as follows: *skn-1* and *skr-1/2* (0.442–0.649), *skn-1* and *daf-16* (0.299–0.542), and *skr-1/2* and *daf-16* (0.238–0.494). (B) Venn diagrams showing numbers of genes overlapping. (C) DAVID functional enrichment analysis of *skr-1/2(RNAi)* up-regulated genes. (D) Linear regression analysis of all genes up-regulated by either *skn-1* or *daf-16(RNAi)* plotted against their fold change with *skr-1/2(RNAi)*; ***P < 0.001 linear regression F-test.
(TIF)

S9 Fig *skr-1/2* is required for juglone and arsenite resistance. Data from three individual trials of survival assays are provided. Representative trials are plotted in Fig 7A and 7B.
(TIF)

S10 Fig Detoxification gene regulation in *skr-1* and *skr-2* mutants. (A) Fold changes in mRNA level of *gst-4*, *gst-10*, *gst-12*, and *gst-30* relative to control in N2 wild-type, *skr-1*(*tm2391*), and *skr-2*(*ok1938*) mutants after exposure to 38 μM juglone for 3 h. ***P<0.001 compared to N2 treated with juglone. (B) mRNA levels of *skr-1* in the *skr-2*(*ok1938*) mutant and *skr-2* in the *skr-1*(*tm2391*) mutant. Values are means plus standard error, *n* = 4 replicates of 200–400 worms.
(TIF)

S11 Fig Overexpression (oe) of *skr-1/2* does not increase expression of SKN-1 regulated genes. (A) Relative *Pgst-4*::*GFP* fluorescence and (B) representative micrographs of worms (QV256) with and without *skr-1/2*(*oe*); note that the extrachromosomal array carries a genomic fragment that includes both *skr-1* and *2*, which are adjacent to each other. *n* = 33 to 56 worms. (C) Fold changes in mRNA level of *skr-1*, *skr-2*, *gst-4*, and *gcs-1* in *skr-1/2*(*oe*) animals relative to control and when treated with or without 38 μM juglone for 3 h. mRNA levels were normalized to *cdc-42*, values are mean plus standard error. *n* = 4 replicates of ~50 worms. ***P<0.001 compared to control. (D) Expression and localization patterns of SKR-1::GFP (QV254) were not obviously affected when treated with 38 μM of juglone for 3h or 5 mM arsenite for 1 h.
(TIF)

S12 Fig SKR-1 is widely expressed throughout the animal. Animals carrying an extrachromosomal array containing SKR-1::GFP and a *Pmyo-2*::*tdTomato* marker (QV254) were fed with either control or *skr-1/2* dsRNA. (A) Shown are DIC images along with fluorescence micrographs taken with GFP and RFP filters. (B) Paired high magnification micrographs of SKR-1::GFP fluorescence and DIC (QV254); the upper right pair is a merger of red and green channels (compare to only GFP in the image immediately below).
(TIF)
