# Supplementary figures and images for: The Skp1 Homologs SKR-1/2 Are Required for the Caenorhabditis elegans SKN-1 Antioxidant/Detoxification Response Independently of p38 MAPK
Source: PLoS Genet. 2016 Oct 24;12(10):e1006361. doi: 10.1371/journal.pgen.1006361 (PMC5077136; doi:10.1371/journal.pgen.1006361)

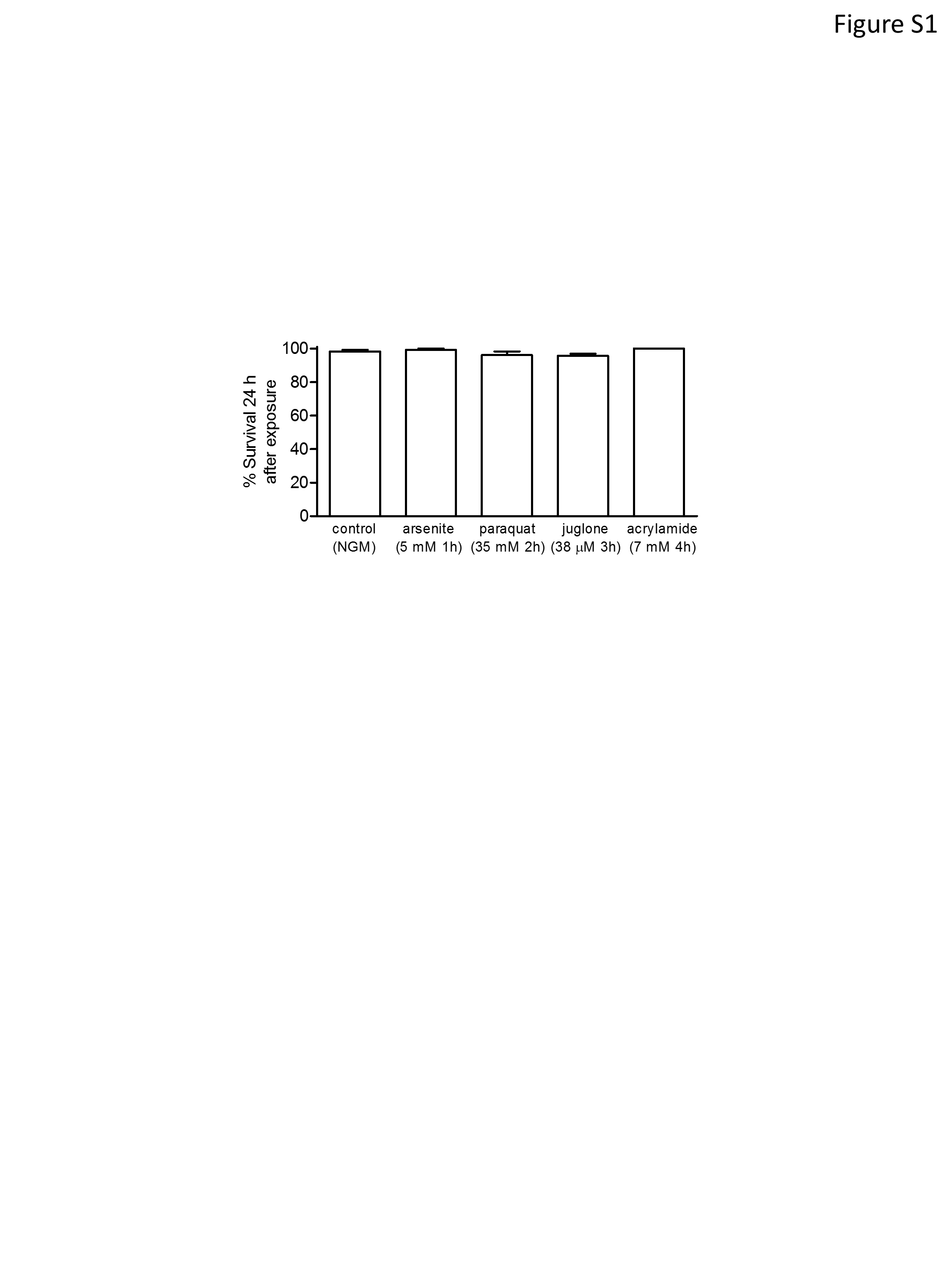

Supplement: S1 Fig — Percentage survival of worms after treatment with each oxidant were scored after 24 h recovery on NGM agar plate seeded with OP50 E. coli. n = 3 trials of 274–402 worms total. (TIF) [file pgen.1006361.s002.TIF]

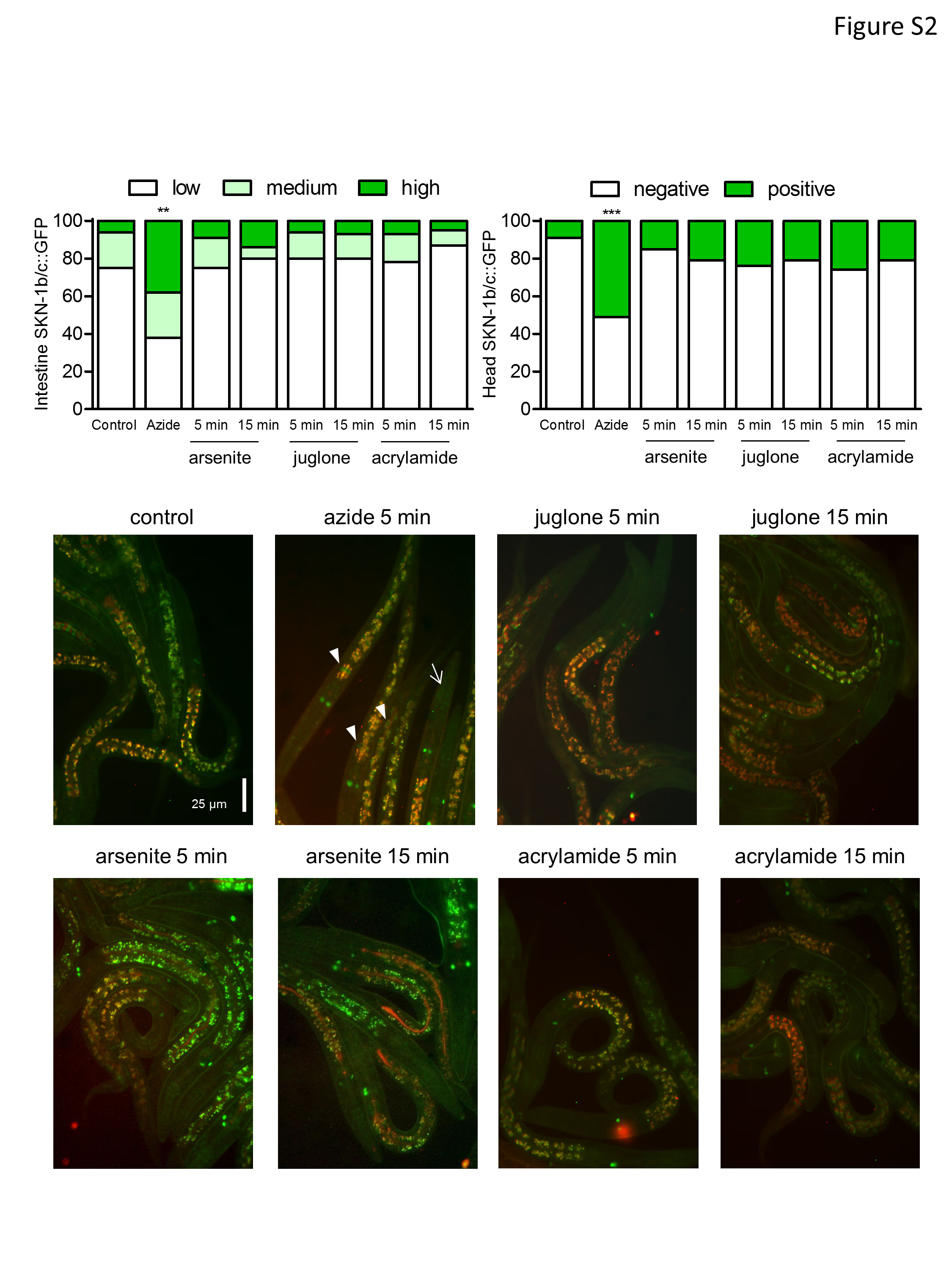

Supplement: S2 Fig — Accumulation of intestinal and head SKN-1b/c::GFP after 5 or 15 min exposure to 2% azide, 5 mM arsenite, 38 μM juglone or 7 mM acrylamide, n = 63–84 worms per condition. ** P<0.01, ***P<0.001 from corresponding controls as determined by the Chi-Square test (bottom). Representative images of SKN-1b/c::GFP. Arrows mark head GFP and arrowheads mark intestinal nuclei. (TIF) [file pgen.1006361.s003.TIF]

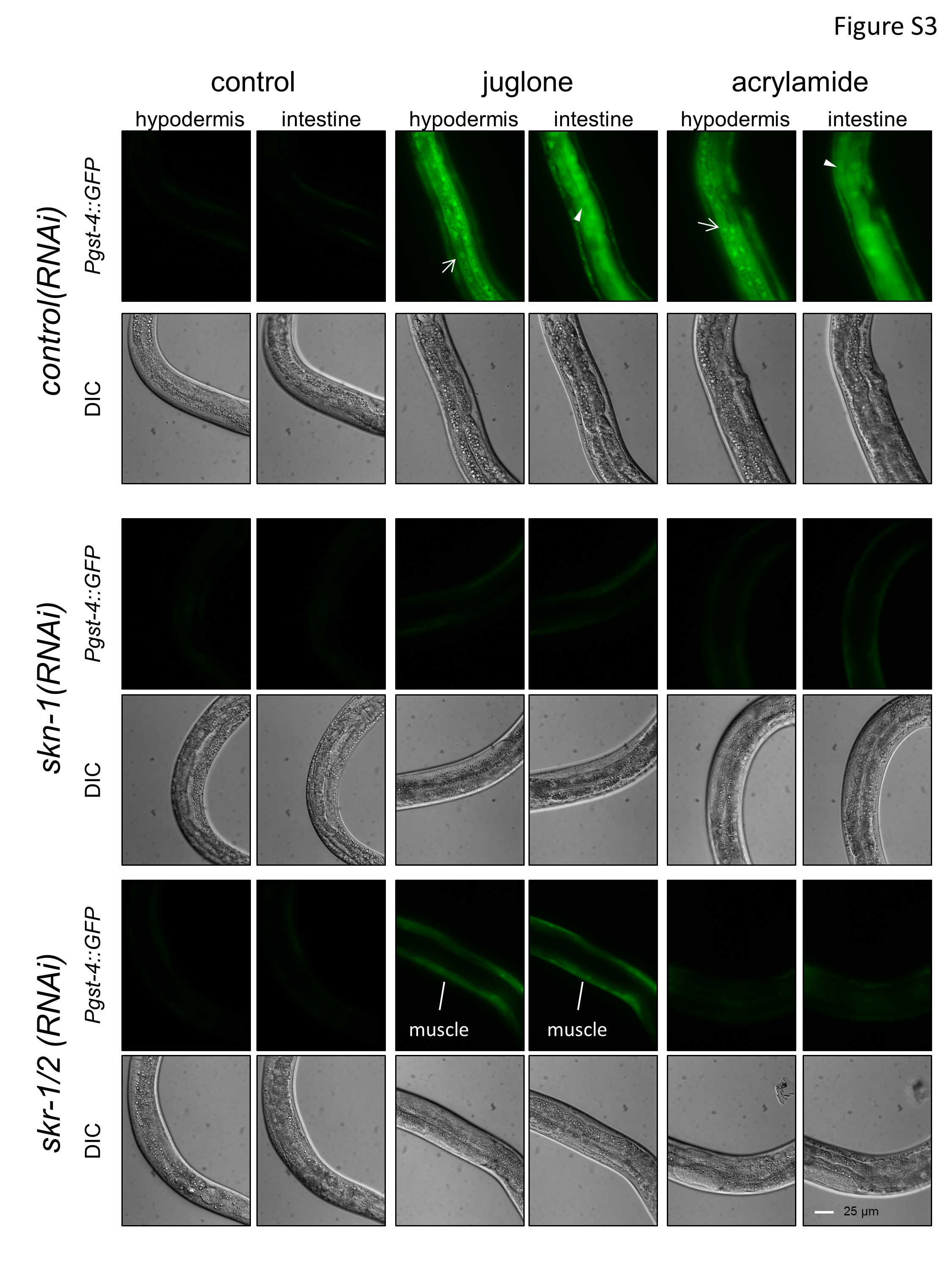

Supplement: S3 Fig — Arrows mark Pgst-4::GFP in hypodermal nuclei, arrowheads mark Pgst-4::GFP in the intestine. (TIF) [file pgen.1006361.s004.TIF]

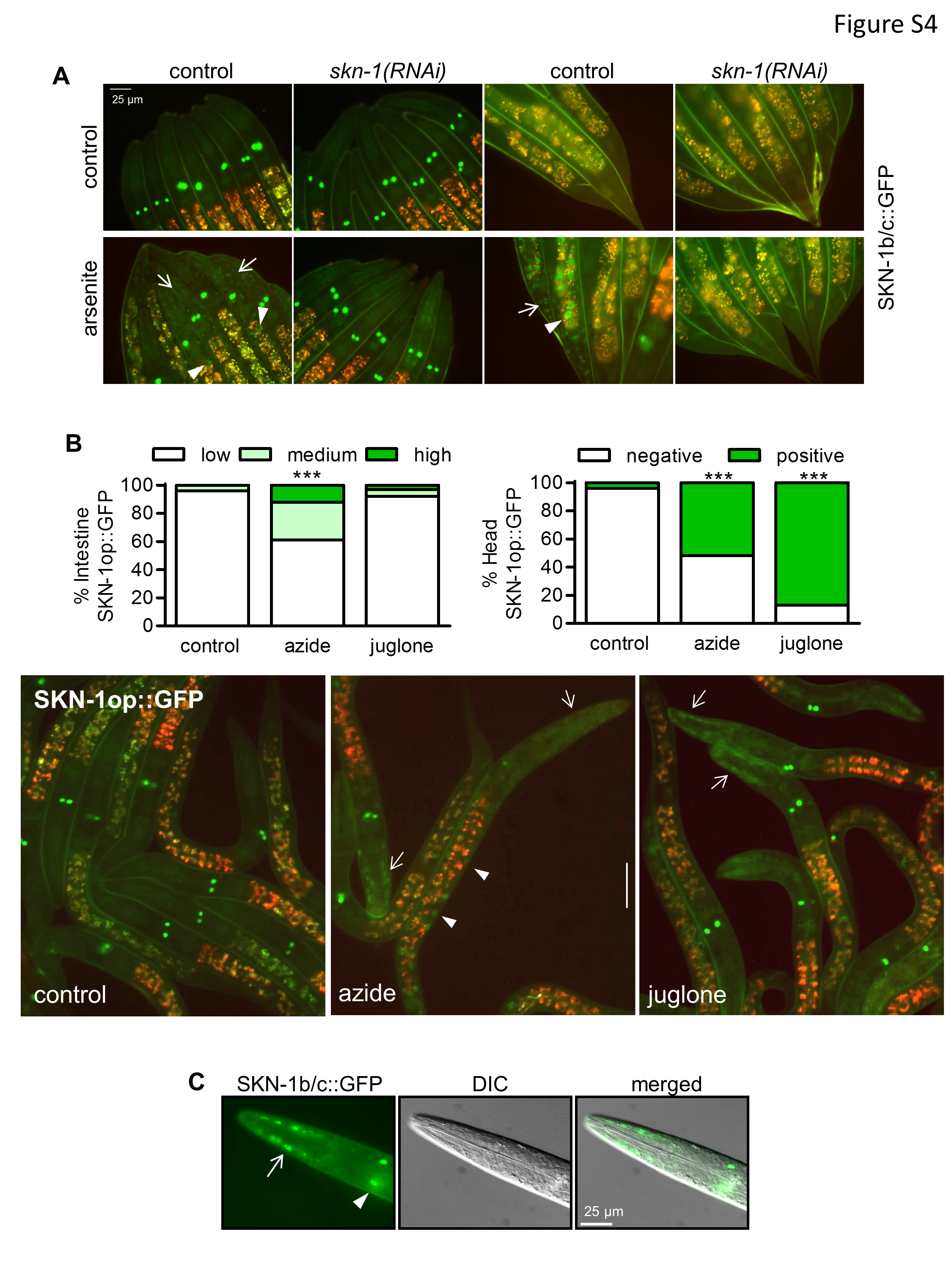

Supplement: S4 Fig — (A) Representative fluorescence micrographs of worms expressing SKN-1b/c::GFP treated with NGM buffer (control) or 5 mM sodium arsenite for 1h fed with control or skn-1(RNAi). (B) Scoring of intestinal and head SKN-1op::GFP and representative fluorescence micrographs. n = 52–71 worms. ***P<0.001 from corresponding controls as determined by Chi-Square tests. Scoring is the same as in Fig 2. (C) Representative fluorescence micrographs and differential interference contrast (DIC) images showing worms expressing SKN-1b/c::GFP in the hypodermis after exposure to 5 mM sodium arsenite for 1 h. Arrows mark head GFP and arrowheads mark ASI neurons. (TIF) [file pgen.1006361.s005.TIF]

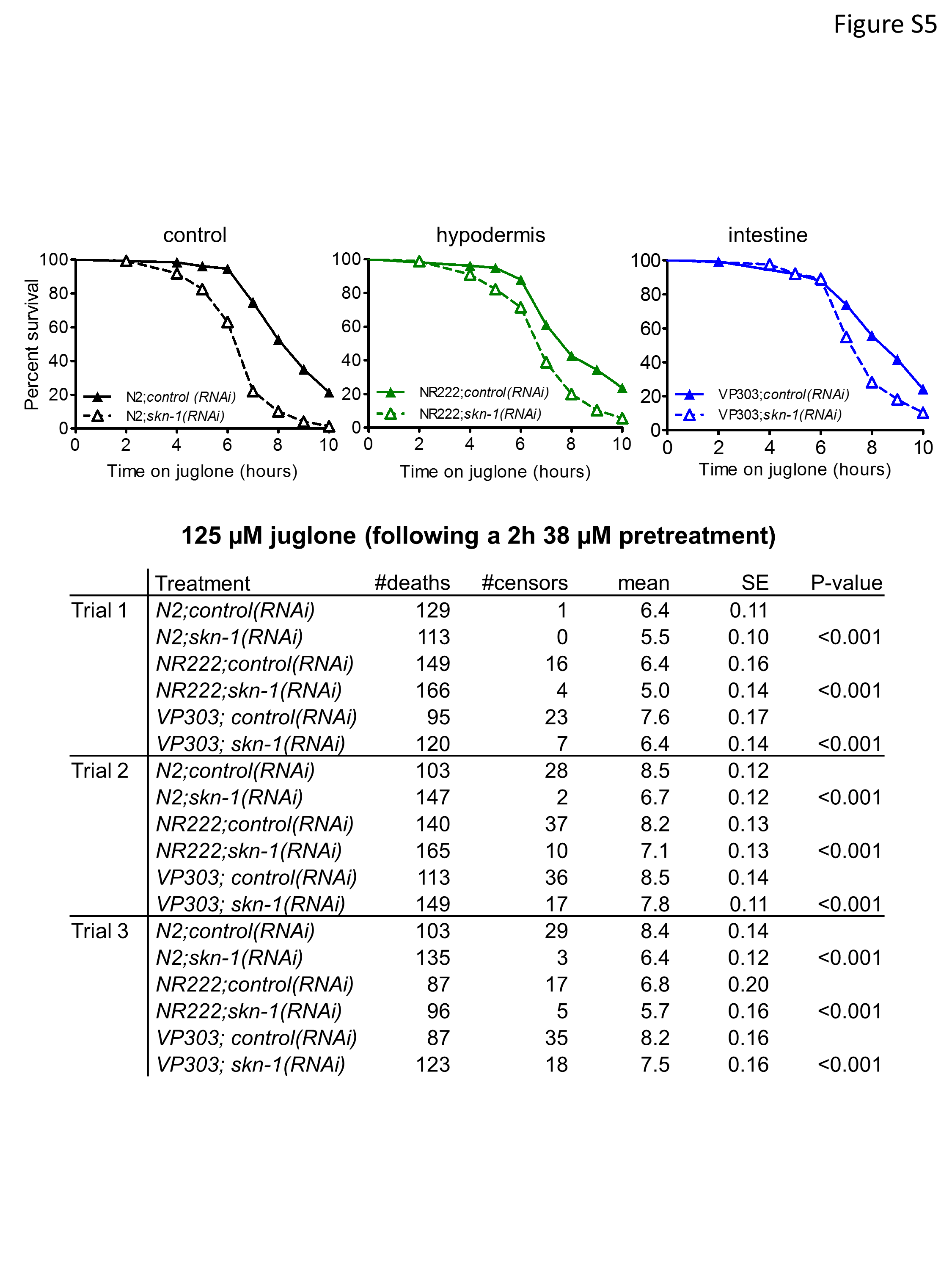

Supplement: S5 Fig — Wildtype and tissue-specific RNAi worms fed with control and skn-1(RNAi) were exposed to 38 μM juglone for 2 h and then the concentration was raised to a total of 125 μM and survival was measured for up to 10 h. Graphs for trial 2 are shown above and a summary of the results for all three trials is show below. skn-1(RNAi) reduced survival in all three strains. (TIF) [file pgen.1006361.s006.TIF]

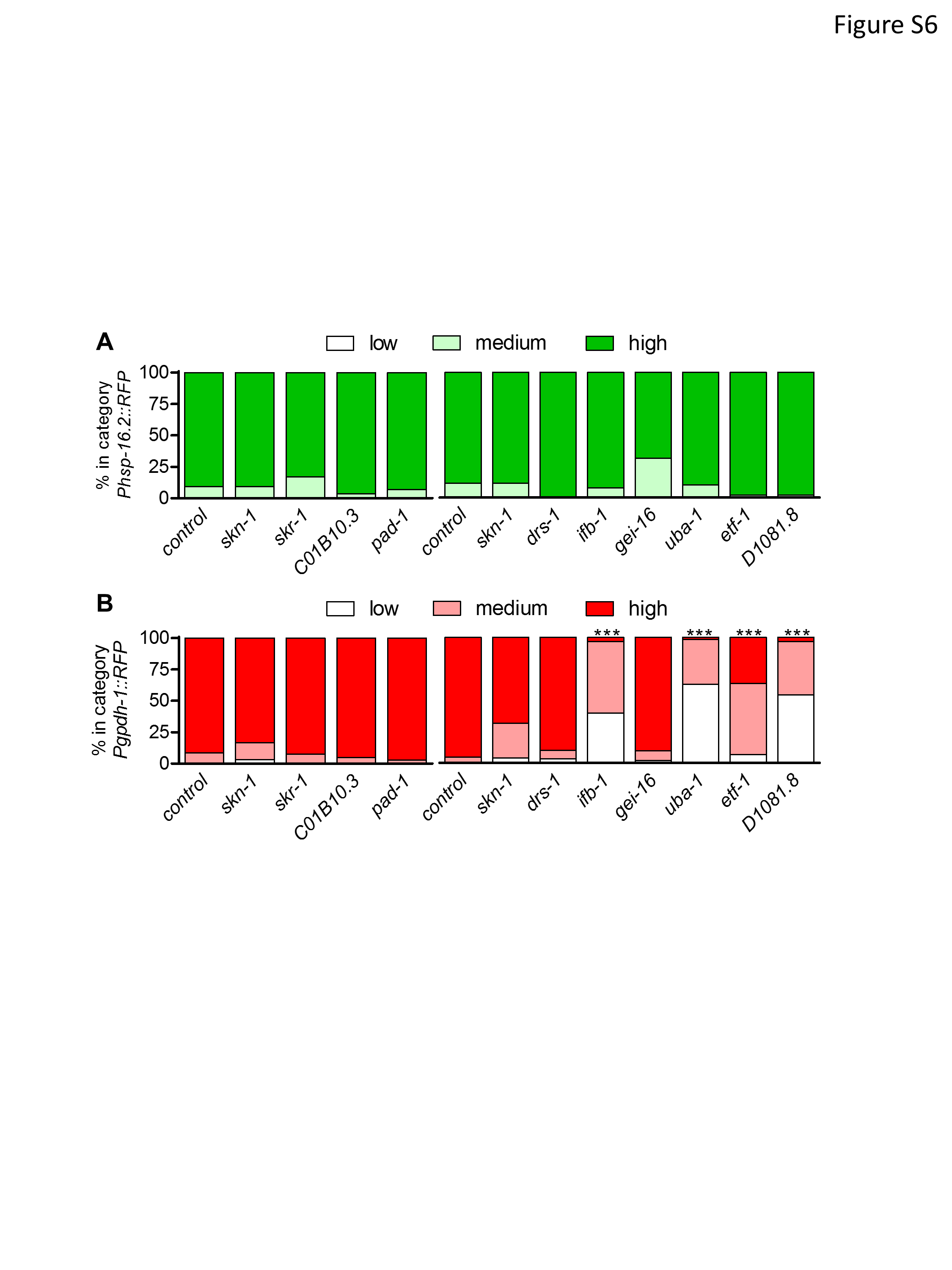

Supplement: S6 Fig — (A-B) Animals integrated with Phsp16.2::GFP or Pgpdh-1::RFP were fed bacteria producing dsRNA and exposed to either heat shock (transfer from 20 to 33°C for 1 h and 5 h recovery for Phsp16.2::GFP) or osmotic stress (transfer from 51 to 250 mM NaCl for 24 h for Pgpdh-1::RFP). Fluorescence was scored as in Fig 4. ***P<0.001 compared to control as determined by Chi-Square tests. (TIF) [file pgen.1006361.s007.TIF]

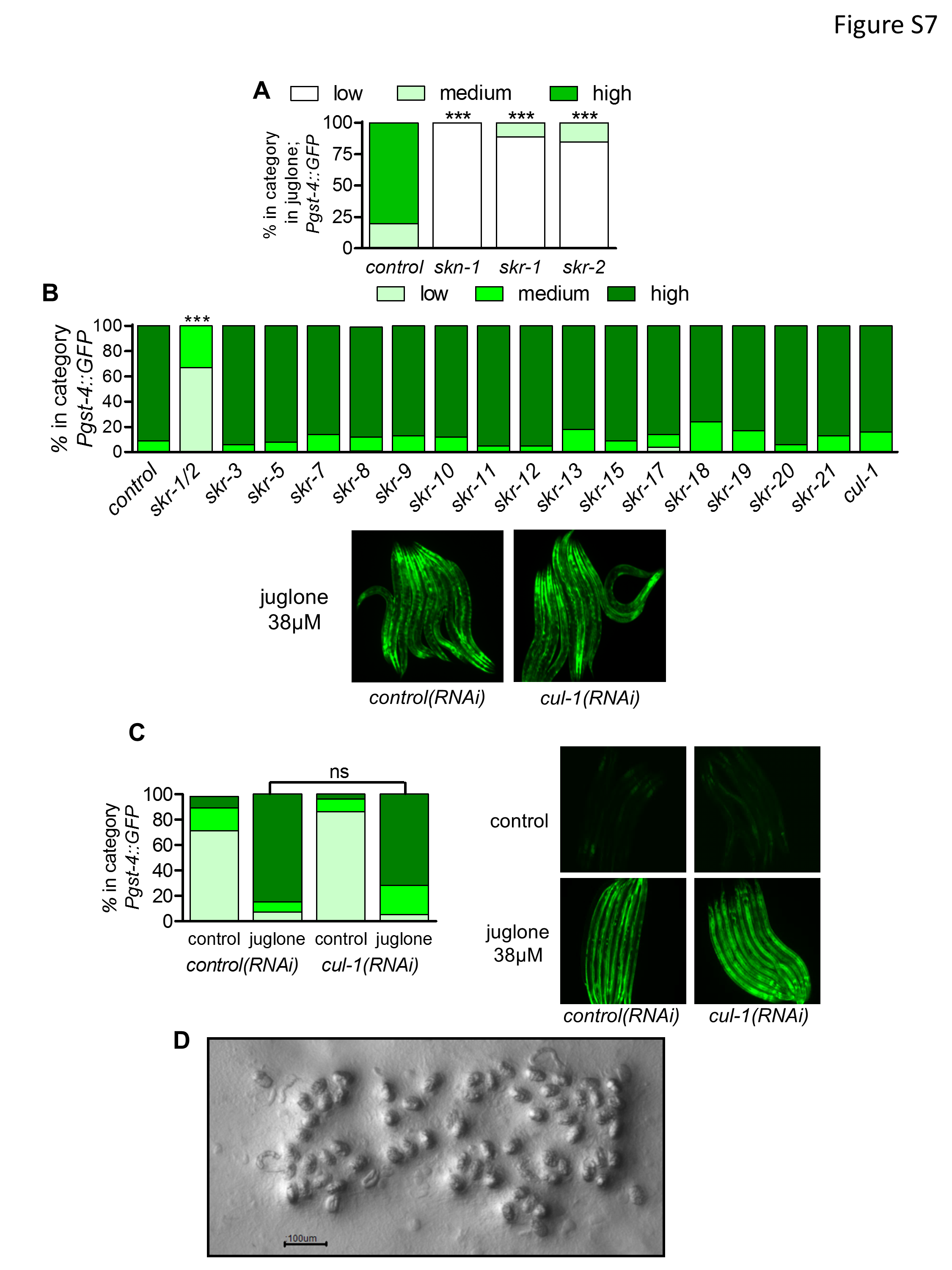

Supplement: S7 Fig — (A) skr-1 and skr-2 RNAi have similar effects on Pgst-4::GFP induced by juglone. (B) RNAi screen against a sub-library of genes functioning within the SCF complex in animals carrying the Pgst-4::GFP reporter after juglone exposure (38 μM for 3 h). ***P<0.001 compared to control as determined by Chi-Square tests. (C) Pgst-4::GFP scoring and representative fluorescence micrographs of eri-1 worms fed with control or cul-1(RNAi) for two generations and exposed to 38 μM juglone for 3 h. (A-C) n = 53–139 worms. (D) High penetrance of embryonic lethal and larval arrest phenotypes are observed in F2 generation of cul-1 RNAi fed eri-1 worms. F1 mothers were allowed to lay eggs and then removed for 24 h before taking an image; note a high number of dead eggs and sick L1 larvae. (TIF) [file pgen.1006361.s008.TIF]

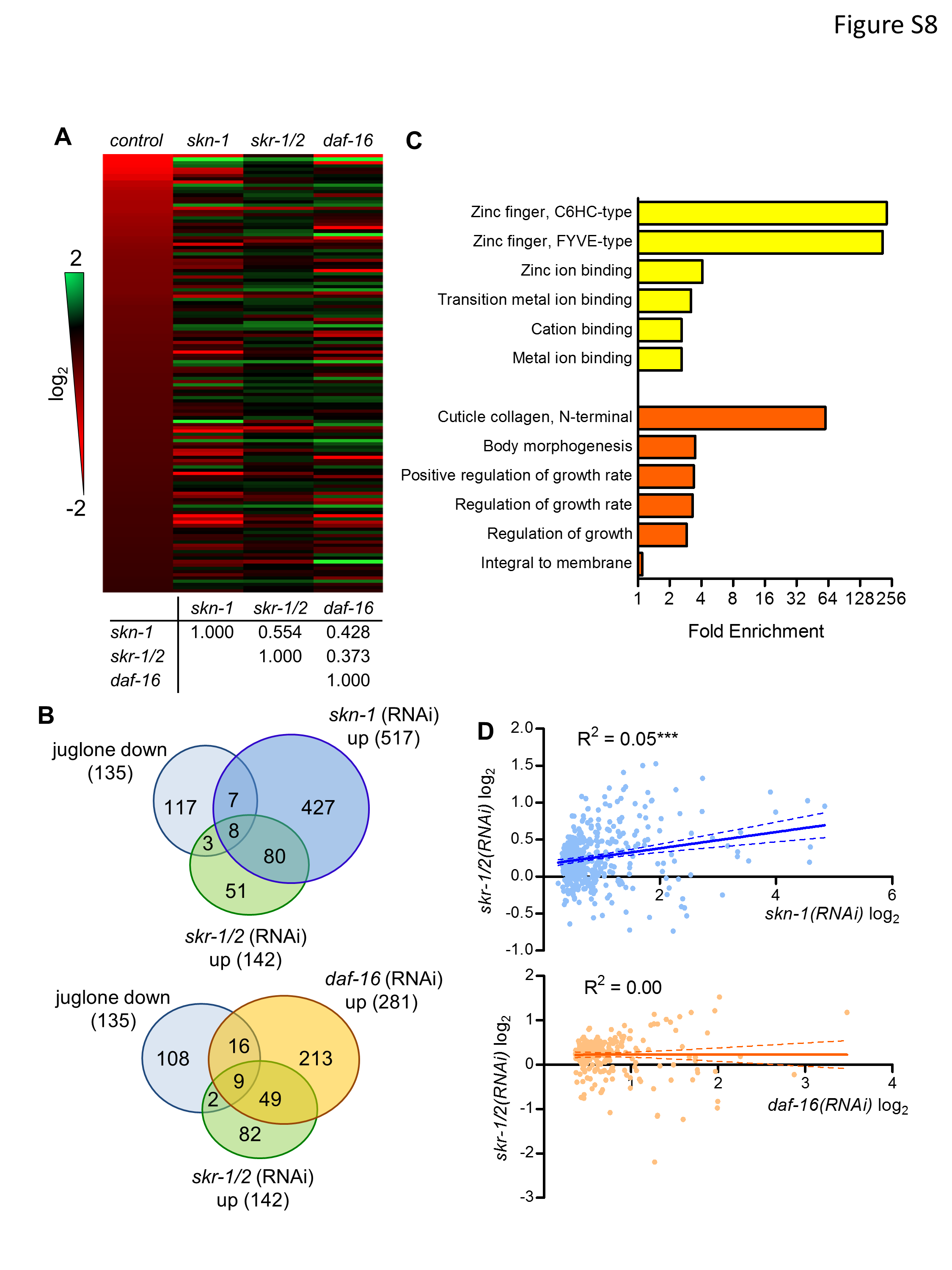

Supplement: S8 Fig — (A) Heat map of fold-changes in 135 genes down-regulated by juglone with their corresponding fold-changes in worms fed with dsRNA for skn-1, skr-1/2 or daf-16. n = 3 replicates of 1,000–2,000 worms. Coefficients of correlation are listed below the heat map and 95% confidence intervals are as follows: skn-1 and skr-1/2 (0.442–0.649), skn-1 and daf-16 (0.299–0.542), and skr-1/2 and daf-16 (0.238–0.494). (B) Venn diagrams showing numbers of genes overlapping. (C) DAVID functional enrichment analysis of skr-1/2(RNAi) up-regulated genes. (D) Linear regression analysis of all genes up-regulated by either skn-1 or daf-16(RNAi) plotted against their fold change with skr-1/2(RNAi); ***P < 0.001 linear regression F-test. (TIF) [file pgen.1006361.s009.TIF]

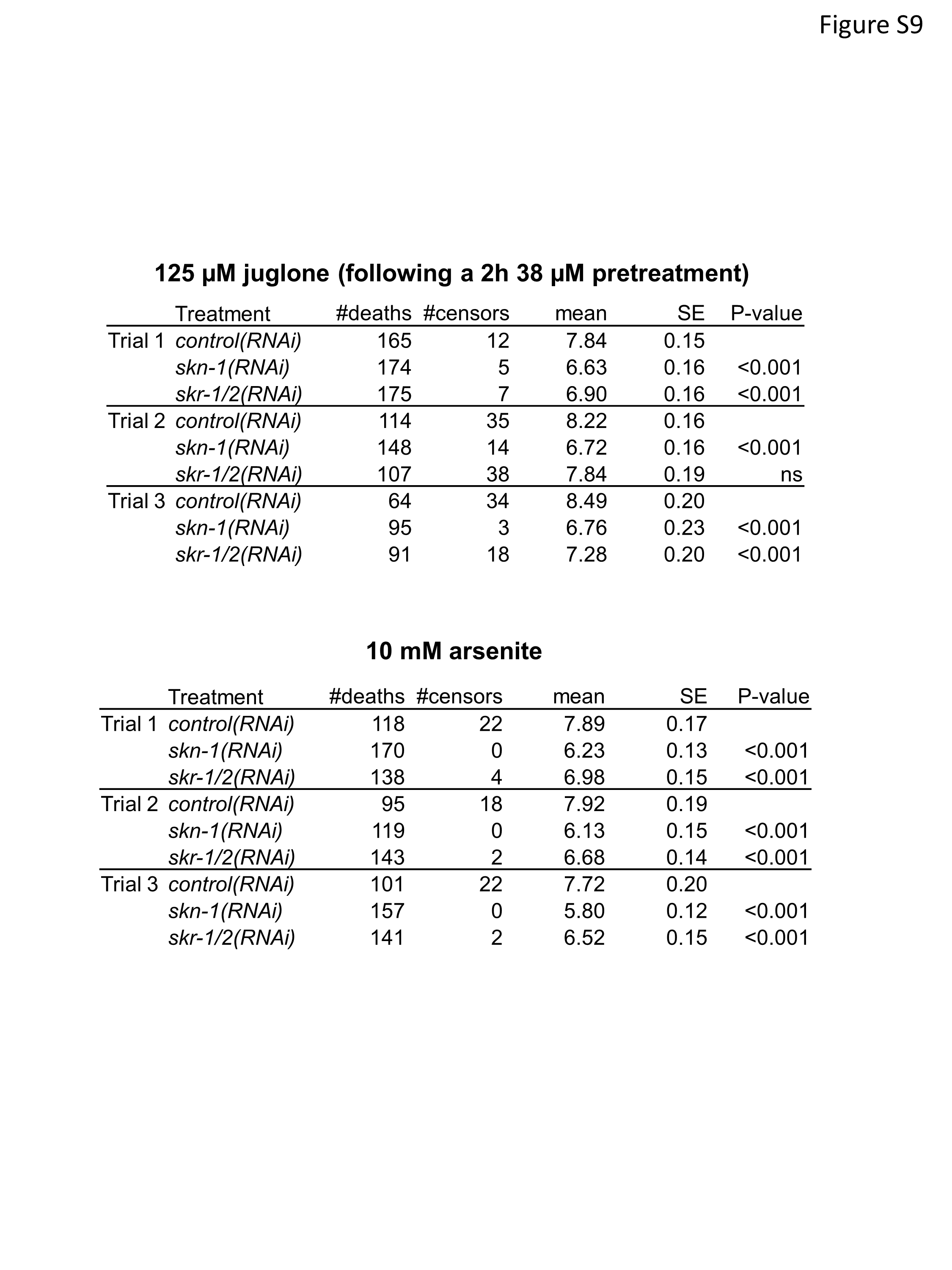

Supplement: S9 Fig — Data from three individual trials of survival assays are provided. Representative trials are plotted in Fig 7A and 7B. (TIF) [file pgen.1006361.s010.TIF]

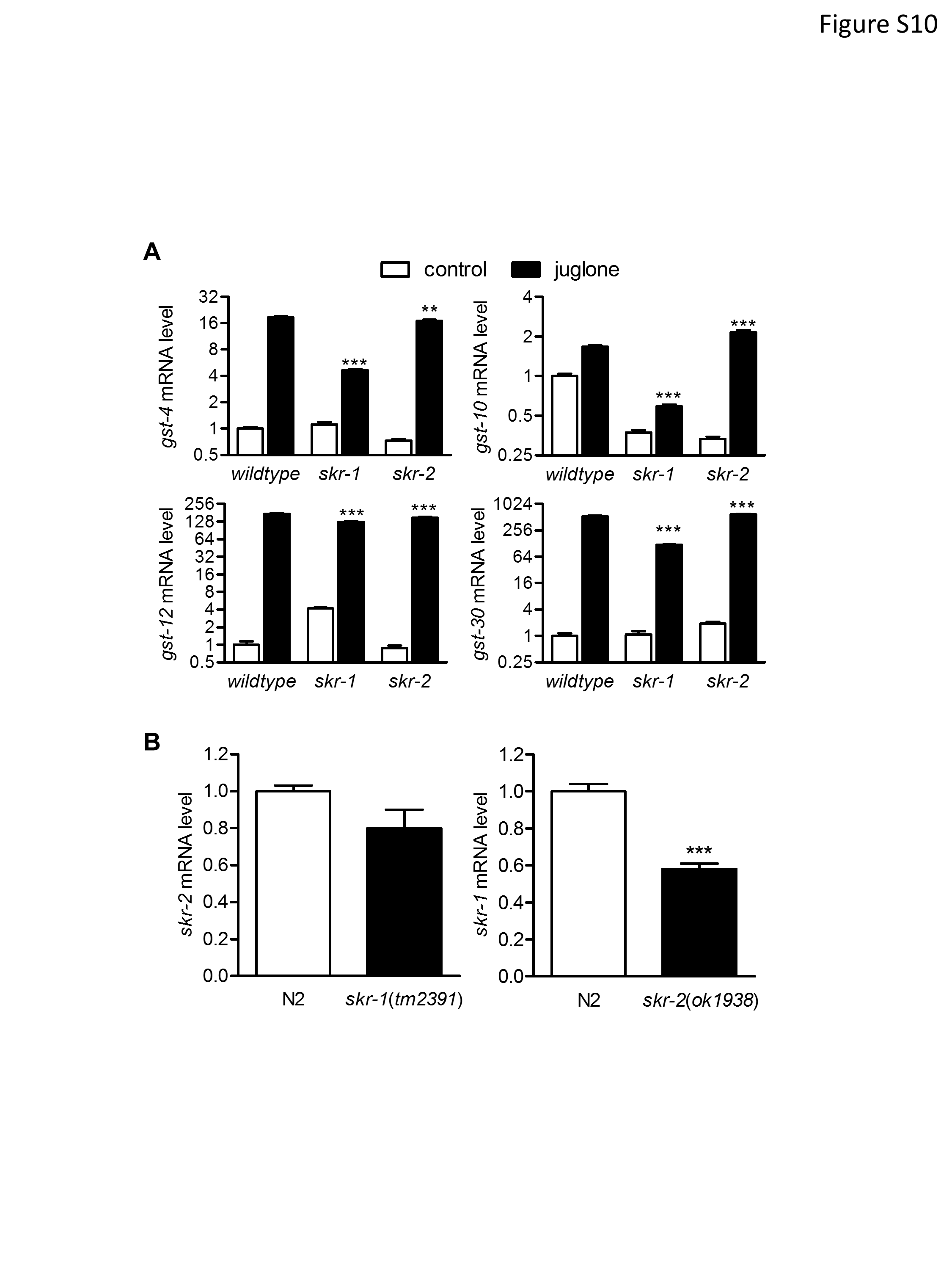

Supplement: S10 Fig — (A) Fold changes in mRNA level of gst-4, gst-10, gst-12, and gst-30 relative to control in N2 wild-type, skr-1(tm2391), and skr-2(ok1938) mutants after exposure to 38 μM juglone for 3 h. ***P<0.001 compared to N2 treated with juglone. (B) mRNA levels of skr-1 in the skr-2(ok1938) mutant and skr-2 in the skr-1(tm2391) mutant. Values are means plus standard error, n = 4 replicates of 200–400 worms. (TIF) [file pgen.1006361.s011.TIF]

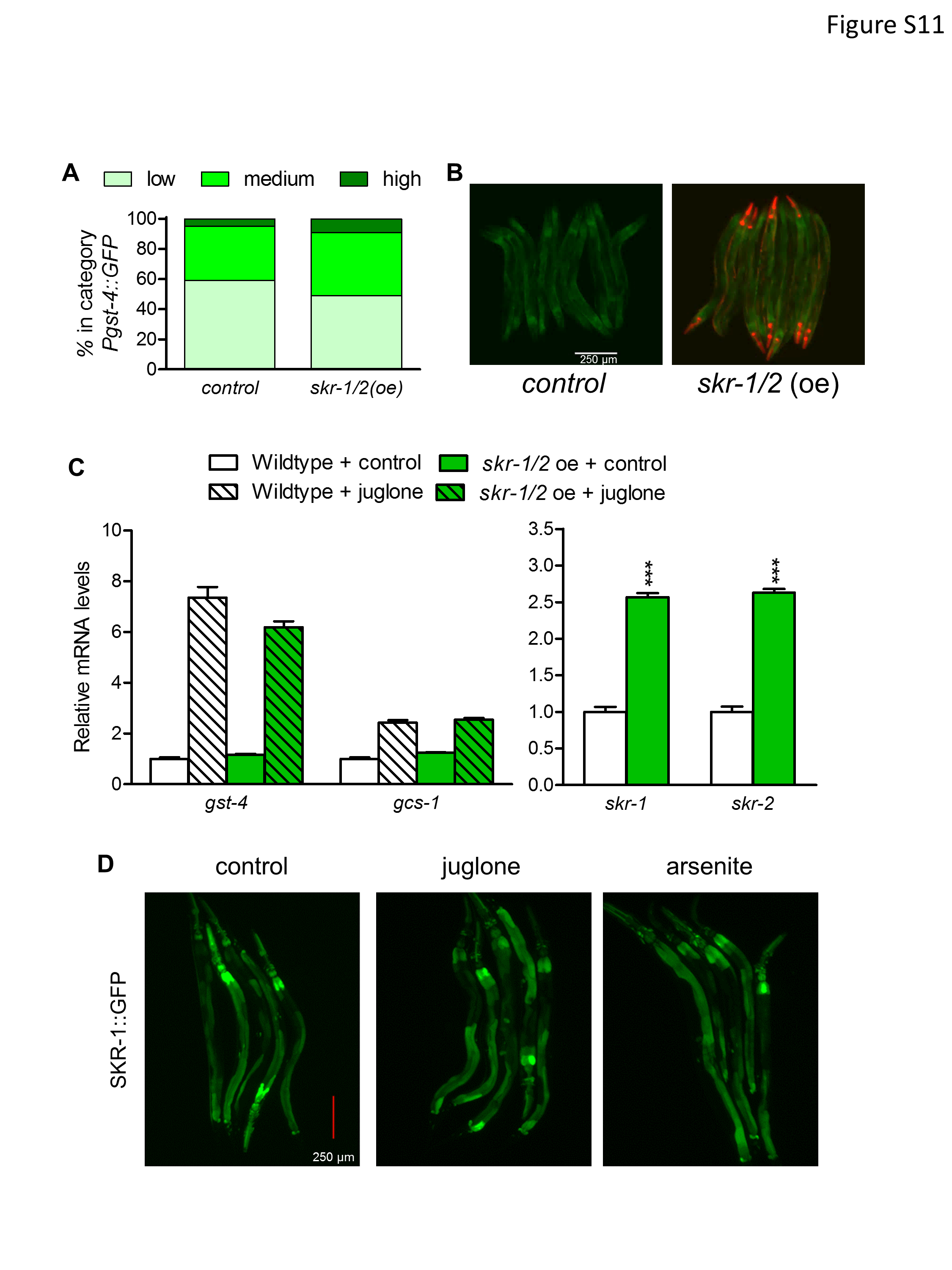

Supplement: S11 Fig — (A) Relative Pgst-4::GFP fluorescence and (B) representative micrographs of worms (QV256) with and without skr-1/2(oe); note that the extrachromosomal array carries a genomic fragment that includes both skr-1 and 2, which are adjacent to each other. n = 33 to 56 worms. (C) Fold changes in mRNA level of skr-1, skr-2, gst-4, and gcs-1 in skr-1/2(oe) animals relative to control and when treated with or without 38 μM juglone for 3 h. mRNA levels were normalized to cdc-42, values are mean plus standard error. n = 4 replicates of ~50 worms. ***P<0.001 compared to control. (D) Expression and localization patterns of SKR-1::GFP (QV254) were not obviously affected when treated with 38 μM of juglone for 3h or 5 mM arsenite for 1 h. (TIF) [file pgen.1006361.s012.TIF]

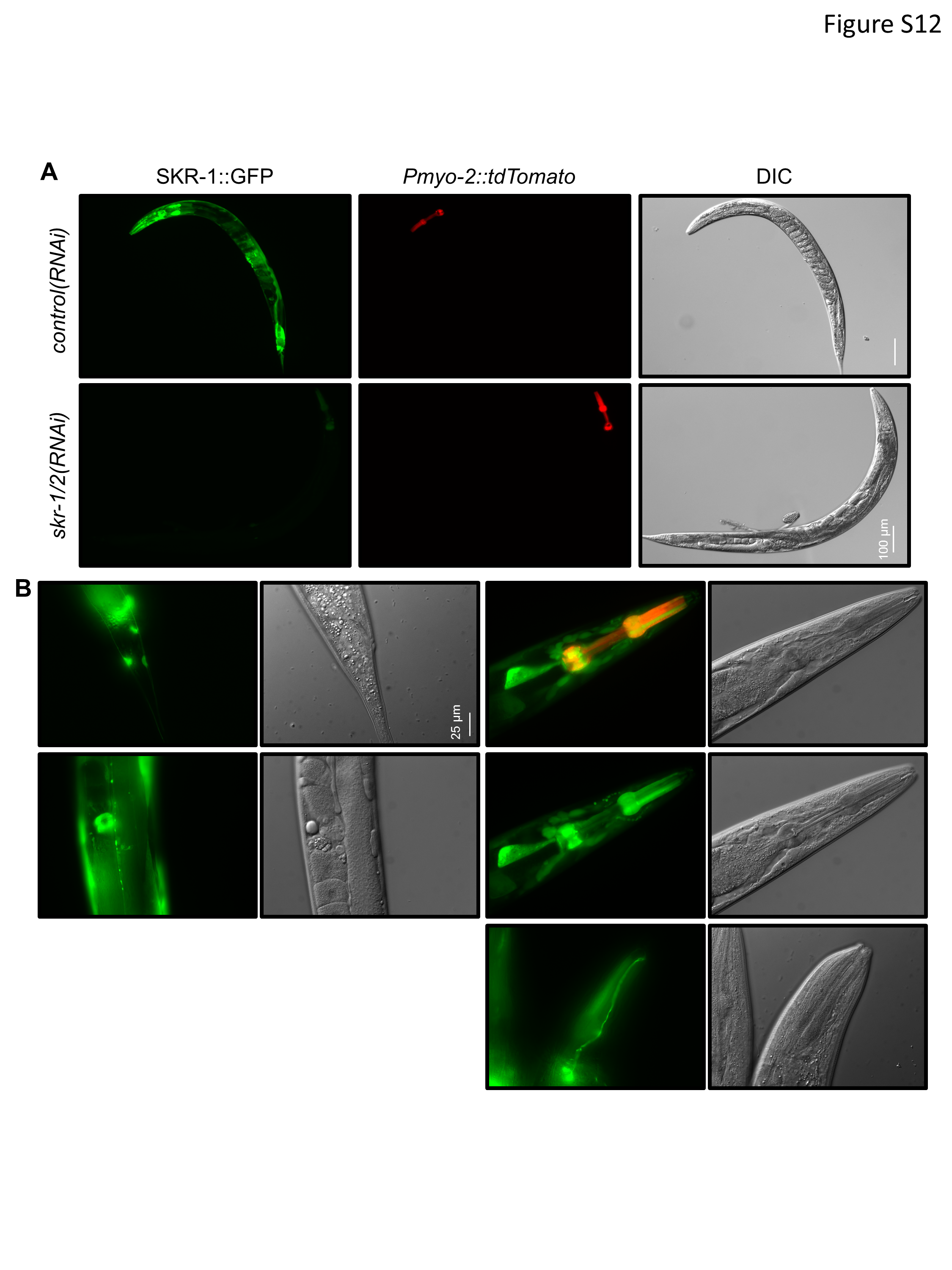

Supplement: S12 Fig — Animals carrying an extrachromosomal array containing SKR-1::GFP and a Pmyo-2::tdTomato marker (QV254) were fed with either control or skr-1/2 dsRNA. (A) Shown are DIC images along with fluorescence micrographs taken with GFP and RFP filters. (B) Paired high magnification micrographs of SKR-1::GFP fluorescence and DIC (QV254); the upper right pair is a merger of red and green channels (compare to only GFP in the image immediately below). (TIF) [file pgen.1006361.s013.TIF]
